# PI3K Signaling and Stat92E Converge to Modulate Glial Responsiveness to Axonal Injury

**DOI:** 10.1371/journal.pbio.1001985

**Published:** 2014-11-04

**Authors:** Johnna Doherty, Amy E. Sheehan, Rachel Bradshaw, A. Nicole Fox, Tsai-Yi Lu, Marc R. Freeman

**Affiliations:** Department of Neurobiology, Howard Hughes Medical Institute, University of Massachusetts Medical School, Worcester, Massachusetts, United States of America; University of Virginia, United States of America

## Abstract

Glial cells are exquisitely sensitive to neuronal injury but mechanisms by which glia establish competence to respond to injury, continuously gauge neuronal health, and rapidly activate reactive responses remain poorly defined. Here, we show glial PI3K signaling in the uninjured brain regulates baseline levels of Draper, a receptor essential for *Drosophila* glia to sense and respond to axonal injury. After injury, Draper levels are up-regulated through a Stat92E-modulated, injury-responsive enhancer element within the *draper* gene. Surprisingly, canonical JAK/STAT signaling does not regulate *draper* expression. Rather, we find injury-induced *draper* activation is downstream of the Draper/Src42a/Shark/Rac1 engulfment signaling pathway. Thus, PI3K signaling and Stat92E are critical *in vivo* regulators of glial responsiveness to axonal injury. We provide evidence for a positive auto-regulatory mechanism whereby signaling through the injury-responsive Draper receptor leads to Stat92E-dependent, transcriptional activation of the *draper* gene. We propose that *Drosophila* glia use this auto-regulatory loop as a mechanism to adjust their reactive state following injury.

## Abstract 2

Activation of glial cells following axon injury is mediated by a positive feedback loop downstream of the glial phagocytic receptor Draper, allowing the strength of the response to match the severity of injury.

## Author Summary

Acute injuries of the central nervous system (CNS) trigger a robust reaction from glial cells—a non-neuronal population of cells that regulate and support neural development and physiology. Although this process occurs after all types of CNS trauma in mammals, how it is activated and its precise role in recovery remain poorly understood. Using the fruit fly *Drosophila melanogaster* as a model, we previously identified a cell surface receptor called Draper, which is required for the activation of glia after local axon injury (“axotomy”) and for the removal of degenerating axonal debris by phagocytosis. Here, we show that regulation of Draper protein levels and glial activation through the Draper signaling pathway are mediated by the well-conserved PI3K and signal transducer and activator of transcription (STAT) signaling cascades. We find that STAT transcriptional activity is activated in glia in response to axotomy, and identify an injury-responsive regulatory element within the *draper* gene that appears to be directly modulated by STAT. Interestingly, the intensity of STAT activity in glial cells after axotomy correlates tightly with the number of local severed axons, indicating that *Drosophila* glia are able to fine-tune their response to neuronal injury according to its severity. In summary, we propose that the initial phagocytic competence of glia is regulated by setting Draper baseline levels (via PI3K), whereas injury-activated glial phagocytic activity is modulated through a positive feedback loop that requires STAT-dependent activation of *draper*. We speculate that the level of activation of this cascade is determined by glial cell recognition of Draper ligands present on degenerating axon material, thereby matching the levels of glial reactivity to the amount of injured axonal material.

## Introduction

Glial cells are extraordinarily sensitive to disruptions in central nervous system (CNS) homeostasis and exhibit an impressive ability to respond to a diversity of neural injuries including hypoxia, chemical insults, and mechanical injury (e.g., axotomy or traumatic brain injury) [1]–[3]. During injury responses, glia exhibit robust changes in gene expression, migrate or extend membranes to sites of trauma, and phagocytose degenerating neuronal debris. Glial reactive responses after injury can be beneficial and promote recovery. For example, glial clearance of degenerating neuronal debris is thought to suppress nervous system inflammation and facilitate remyelination [4]–[8]. However, glia can also exacerbate damage in the CNS by driving inflammation through the release of pro-inflammatory cytokines and actively destroying healthy cells [9]–[14]. Whether reactive gliosis is ultimately more beneficial or harmful to the nervous system remains an open question and is likely context dependent. However, mounting evidence points to reactive glial cells as an excellent target for therapeutically modifying CNS disorders [15].

Reactive glial cells have been studied in a variety of neurodegenerative diseases and injury models but surprisingly little is known about how glial sensitivity to neuronal health is initially established in the healthy brain, or how glial perception of neuron-derived “injury signals” is translated into dynamic reactive responses after trauma. A wealth of studies indicate that reactive gliosis is not an all-or-none response, but rather includes a wide range of graded responses that correlate with the severity of nervous system injury [15]. The ability of glia to “measure” the severity of trauma in the nervous system and respond accordingly implies a tight functional coupling between signaling pathways sensing neural injury and those executing glial reactive responses [16]. In some cases reactive glia can modulate the intensity of reactive gliosis in an autocrine way. For example, focal demyelination leads to activation and release of endothilin-1 (ET-1) from astrocytes, which in turn can signal back to astrocytes (through ET-1 receptors) and further enhance astrocyte cellular hypertrophy, proliferation, and GFAP expression [17]–[22]. However, the initial injury signal which activates ET-1 expression remains to be identified. An intriguing possibility is that so-called “eat me” cues on degenerating neuronal debris directly activate receptors on glial cells, which in turn modulate initiation of reactive gliosis, but evidence for such molecular regulation is lacking.

We have previously shown that Draper, a glial-expressed immunoreceptor-like molecule, is a key surface receptor required for glial engulfment of degenerating axons after axotomy in the adult *Drosophila* brain [23]–[25]. Draper is the *Drosophila* ortholog of CED-1, an engulfment receptor that is essential for engulfment of cell corpses in *Caenorhabditis elegans* [26]. Within hours after axotomy of *Drosophila* antennal olfactory receptor neuron (ORN) axons, Draper protein, and mRNA levels are dramatically increased in glia surrounding degenerating axons. Glial membranes are then recruited to severed axons, glia phagocytose axonal debris, and ultimately glia return to a resting state [24],[27]. Loss of Draper function blocks all glial morphological and molecular responses to axonal injury and axonal debris lingers in the brain for the life of the animal. Similar phenotypes have been observed when the components of the Src-family kinase signaling cascade that acts downstream of Draper (i.e., Shark and Src42a) are eliminated specifically from glial cells [25]. These data argue that Draper acts very early in the activation of *Drosophila* glia after axonal injury, perhaps even in the recognition of cues presented by engulfment targets like degenerating axons. Recently the mammalian orthologs of CED-1/Draper, MEGF10, and Jedi, have been implicated in satellite glial engulfment of neuronal cell corpses in developing mouse dorsal root ganglia [28] and MEGF10 has been shown to engulf pruned synapses in the postnatal dorsal lateral geniculate nucleus during activity-dependent synaptic refinement [29]. Thus, Draper/MEGF10/Jedi engulfment signaling appears to be a conserved feature of glial cells in evolutionarily distant species.

To further explore the molecular mechanisms by which glial cells establish competence to respond to axonal injury and dynamically regulate reactive responses, we performed an *in vivo* RNAi screen for novel signaling molecules required for glial engulfment of degenerating ORN axons in *Drosophila*. We identified PI3K signaling and the Stat92E transcription factor as important regulators of glial Draper expression. Interestingly, both of these pathways were necessary for the expression of Draper in the resting, uninjured brain. However, while PI3K signaling was dispensable for injury-induced up-regulation of Draper, we found that Stat92E was necessary in glial cells for both injury-induced up-regulation of Draper and clearance of degenerating axonal debris. Surprisingly, we find that Stat92E acts downstream of Draper to activate transcription. We propose a simple model for glial activation after axotomy whereby Draper signaling is stimulated in a graded fashion according to the level of axonal debris (i.e., the severity of axonal injury), which in turn promotes Stat92E-dependent changes in glial gene expression in a way that is proportional to the strength of Draper pathway signaling. Such a mechanism places levels of glial activation directly downstream of the total amount of axonal debris present in the adult brain.

## Results

### Glial PI3K Signaling Modulates Levels of the Engulfment Receptor Draper in the Healthy Brain

**Figure 1:**
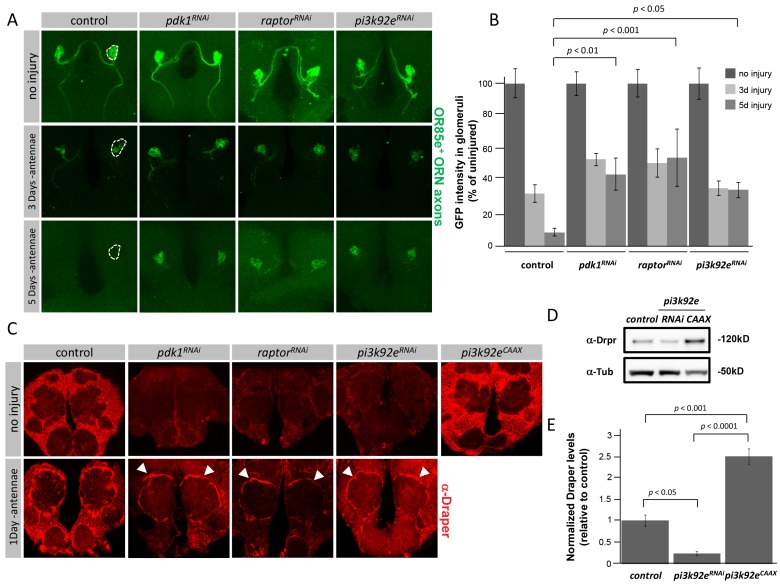
PI3K signaling modulates basal levels of glial Draper expression. (A) Z-stack confocal images of OR85e^+^ axons labeled with GFP; *repo-Gal4* was used to drive *UAS-pdk1^RNAi^, UAS-pi3k92e^RNAi^, and UAS-raptor^RNAi^* in a background containing the temperature sensitive Gal4 repressor, Gal80^ts^; Uninjured, 3 days, and 5 days after maxillary palp ablation are shown. Dotted lines indicate 85e^+^ glomerulus used for quantification of GFP remaining. (B) Quantification of GFP intensities in 85e^+^ glomeruli from (A), *p*-values were calculated using one-way ANOVA and Fisher's LSD post-hoc analysis. Error bars represent standard error of the mean (SEM). (C) Single slice confocal images of adult brains; Draper antibody stains in control (*OR85e-GFP,Gal80^ts^*/+; *repo-gal4*/+) and RNAi backgrounds (*OR85e-GFP,Gal80^ts^/+; repo-gal4/UAS-pdk1^RNAi^*; *UAS-pi3k92e^RNAi^*, *OR85e-GFP,Gal80^ts^/+; repo-gal4/+*; and *OR85e-GFP,Gal80^ts^/UAS-raptor^RNAi^; repo-gal4/+*). Uninjured and one day after antennal ablation are shown. (D) Western blot analysis of Draper protein levels in ∼3 adult central brain regions of control (*OR85e-GFP,Gal80^ts^*/+; *repo-gal4*/+), *pi3k92e^RNAi^* (*UAS-pi3k92e^RNAi^*, *OR85e-GFP,Gal80^ts^/+; repo-gal4/+)*, and *pi3k92e^CAAX^* (*UAS-pi3k92e^CAAX^*, *OR85e-GFP,Gal80^ts^/+; repo-gal4/+*) genotypes. α-Tubulin was used as a loading control. Full blot located in Figure S1. (E) Quantification of Draper protein levels shown on Western blot after normalization to Tubulin, *n* = 3, *p*-values were calculated using one-way ANOVA followed by Tukey's post hoc test.

In order to identify new glial engulfment genes we performed an *in vivo* RNAi screen using a previously established assay [24]. Briefly, ∼300 candidate engulfment genes were knocked down specifically in glia by driving *UAS*-regulated RNAi constructs with the glial specific *repo-Gal4* driver. To assay the ability of glia to clear degenerating axonal debris we labeled a subset of maxillary palp ORNs with green fluorescent protein (GFP), severed the axons by surgically removing the maxillary palps, and assayed for clearance of GFP-labeled ORN axonal debris at various timepoints. Interestingly, we found glial-specific knockdown of *pi3k92e*, *raptor*, or *pdk-1*—key components of the phosphoinositide 3-kinase (PI3K) signaling pathway—led to a decrease in glial clearance of axonal debris 5 days after axotomy (Figure 1A and 1B; Data S1). Similar results were found when we drove glial expression of a dominant-negative version of PI3K92e (*UAS-pi3k92e^DN^*; Figure S1; Data S2). We note that while axon clearance was delayed in these backgrounds, ultimately all axonal debris was cleared from the brain 7–10 days after axotomy (unpublished data), indicating that glia exhibited a delay in clearance rather than a blockade.

To further explore the cellular basis for this delayed axon clearance phenotype, we assayed glial expression of the engulfment receptor Draper. Surprisingly, glial knockdown of *pi3k92e*, *raptor*, or *pdk1*, or over expression of *pi3k92e^DN^* reduced Draper expression significantly in the uninjured brain (Figures 1C and S1; Data S3). Reductions in Draper protein levels were confirmed and quantified on Western blots (Figures 1D, 1E, and S1; Data S3). Reciprocally, we found that over-expression of a constitutively activate version of PI3K92E (*UAS-pi3k92e^CAAX^*) in glia led to a dramatic increase in Draper levels in uninjured brains (Figure 1C–1E; Data S3). Thus, Draper expression was tightly correlated with glial PI3K signaling levels in the healthy uninjured brain. To our knowledge this is the first pathway shown to modulate the establishment of Draper expression levels in glia.

While maxillary palps house ∼60 ORN cell bodies and ablation of maxillary palps results in a modest increase in Draper protein expression, more severe injury of ORN axons by removal of antennae (which house ∼600 ORN cell bodies) results in a dramatic up-regulation of Draper protein and mRNA levels [24],[27]. To determine whether this response was normal when the PI3K pathway was compromised, we ablated antennae in control, *pi3k92e^RNAi^*, *raptor^RNAi^*, and *pdk1^RNAi^* backgrounds. Despite the fact that knockdown of *pi3k92e*, *raptor*, or *pdk1* significantly reduced Draper levels in the brains of uninjured control animals, we found that antennal ORN axotomy induced significant up-regulation of Draper levels in glia surrounding the antennal lobe (Figure 1C). While it is possible that this injury-induced Draper up-regulation could be the result of incomplete RNAi mediated knockdown of components of the PI3K signaling pathway, on the basis of the consistency in results among the different components of the pathway we favor the notion that basal levels of Draper (i.e., those present in the healthy brain) are regulated by PI3K signaling and injury-induced up-regulation of Draper is regulated by alternate signaling pathways.

### The Draper Locus Contains an Enhancer Element That Is Responsive to Axonal Injury through Stat92E

**Figure 2:**
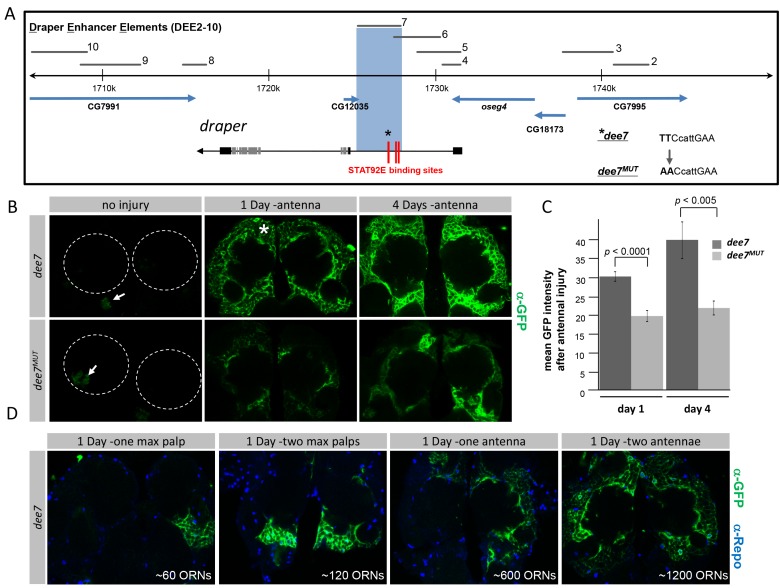
The *draper* locus contains a Stat92E-regulated injury responsive element. (A) Schematic representation of the *draper* locus and regions used to generate DEEs 2–10. Blue box highlights *dee7* region located in the first intron of the *draper* gene. Red lines indicate Stat92E binding sites in *dee7*. Asterisk indicates binding site in *dee7^MUT^* containing two point mutations. (B) Single slice confocal images of antennal lobe regions; *dee7-Gal4* or *dee7^MUT^-Gal4* driving two copies of *UAS-mCD8::GFP (dee7>2XmCD8::GFP, dee7^MUT^>2XmCD8::GFP)*. Uninjured, one day after antennal ablation and four days after antennal ablation are shown. Dashed circles outline antennal lobes, the site of injury. Asterisk indicates cortex glia responding to axotomy. (C) Quantification for (B), *p*-values were calculated using Student's *t* test, *n.s.*, not significant. Error bars represent SEM. (D) Single slice confocal images of antennal lobe regions stained with α-GFP and α-Repo; *dee7-Gal4* was used to drive two copies of *UAS-mCD8::GFP*. Removal of one maxillary palp, two maxillary palps, one antenna, and two antennae are shown one day after injury.

Our observations that basal and injury induced Draper expression were controlled by distinct molecular pathways prompted us to attempt to identify *draper* gene enhancer elements responsible for establishing basal levels of *draper* expression in adult brain glia, and/or increasing *draper* expression specifically after ORN axotomy. We focused our search on an ∼40 kb region centered around the *draper* locus (Figure 2A). We cloned nine different potential *draper* enhancer elements (termed *dee2-dee10*) from 5′, intronic, or 3′ regions of the *draper* gene into the *Gal4*-based *pBGW* vector [30] and inserted these elements into identical genomic locations (Figure 2A). Each *dee-Gal4* line was then used to drive two copies of *UAS-mCD8::GFP in vivo* and expression patterns were examined in the adult brain before and after injury. No expression in ensheathing or cortex glia was observed with any of the enhancer element lines in the healthy, uninjured brain (unpublished data). We therefore failed to identify any single enhancer element that was capable of driving glial expression of reporters in the adult brain in a pattern similar to endogenous Draper protein. This observation suggests that PI3K-dependent regulation of Draper levels might be governed by an enhancer element some distance from the *draper* gene, requires the convergent activity of multiple enhancers along the *draper* gene, or could be controlled through post-transcriptional mechanisms.

To determine whether any of these potential DEEs were responsive to axotomy, we ablated antennae or maxillary palps and assayed reporter activation in glia one day after injury. We did not observe glial expression after axonal injury with *dee2-6-* or *dee8-10-Gal4* lines (unpublished data). However, one day after antennal ORN axotomy, we observed a striking increase in glial expression of mCD8::GFP in the *dee7-Gal4* reporter background, and GFP levels were further increased four days after axon injury (Figure 2B). We note that in uninjured animals we observed low level expression of this element in astrocyte-like glia, which were randomly distributed in the neuropil (Figure 2B) but it did not drive GFP expression prior to axon injury in ensheathing or cortex glia, those adult brain glia which normally express Draper [23]. Interestingly, not only was injury-induced reporter expression strong in ensheathing glia surrounding the antennal lobe—those glia that normally engulf degenerating ORN axons [23]—but the reporter expression also increased robustly in cortex glia throughout the brain (Figure 2B and 2D). This widespread activation of the reporter supports the notion that glia, even at locations distant from the injury site, can respond molecularly to axonal damage. Notably, we found the activation of the *dee7-Gal4* element appeared to scale with the severity of the axonal injury: when we ablated one maxillary palp (∼60 ORNs), two maxillary palps (∼120 ORNs), one antenna (∼600 ORNs), or both antennal (∼1,200 ORNs), we observed a correlated increase in *dee7-Gal4*-driven mCD8::GFP (Figure 2D). We further note that ablation of maxillary palps, whose axons are found in the maxillary nerve and ventro-medial regions of the antennal lobe, resulted in a much reduced and more localized increase in *dee7-Gal4*-driven mCD8::GFP in cortex glia located in the ventral region of the antennal lobe (Figure 2D).

Sequence analysis of the 2619 bp *dee7* element led to the discovery of three consensus Stat92E binding sites (TTC3n/4nGAA) [31], the sole member of the signal transducer and activator of transcription (STAT) family of molecules in *Drosophila* [31],[32]. Of these three Stat92E sites, two were also present in *dee6-Gal4*, which was not responsive to axonal injury (Figure 2A). We mutated the Stat92E binding site specific to the *dee7-Gal4* element, integrated this *dee7^MUT^*-*Gal4* construct into the same genomic location used for the previously generated reporter lines, and examined its responsiveness to axonal injury. While baseline levels of mCD8::GFP expression were similar to *dee7-Gal4*, *dee7^MUT^-Gal4* exhibited an ∼30%–40% decrease in the injury-induced expression of mCD8::GFP at both 1 and 4 days after axotomy (Figure 2B and 2C; Data S4). In contrast, simultaneous mutation of the two other Stat92E binding sites within the *dee7* element had no effect on transcriptional activation of the *dee7-Gal4* reporter (Figure S2A–S2C; Data S5). From these data we conclude that *dee7* contains a glial transcriptional regulatory element that is responsive to axonal injury, and our data suggest that at least one Stat92E binding site is required for maximal activation of this element after axotomy.

### Glial Loss of STAT92E Suppresses Clearance of Axonal Debris and Eliminates Expression of Draper

**Figure 3:**
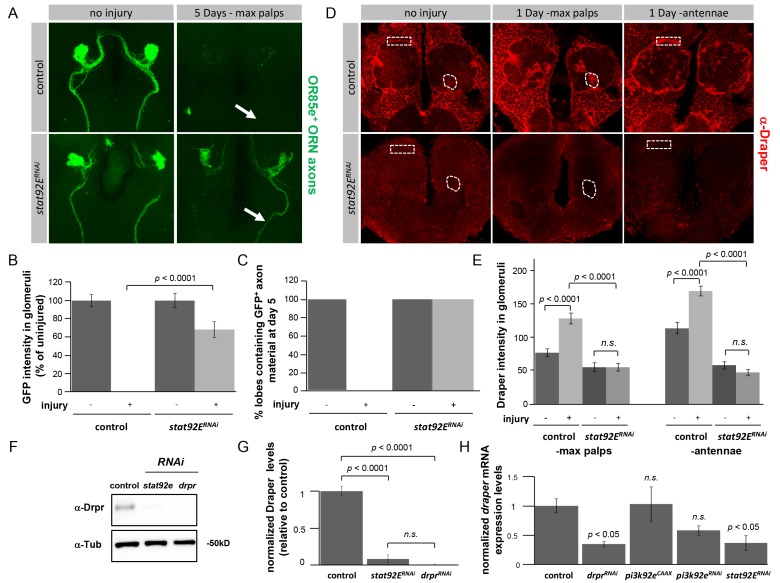
Stat92E functions in glia to promote axon clearance and regulate Draper expression. (A) Z-stack confocal images of OR85e^+^ axons labeled with GFP in control (*OR85e-GFP/+; repo-gal4/+*) or *stat92e^RNAi^* (*stat92e^RNAi^*/*OR85e-GFP;repo-Gal4*/+) backgrounds. Uninjured and 5 days after maxillary palp ablation are shown. Arrows indicate position of maxillary nerves. (B) Quantification of GFP intensities in 85e^+^ glomeruli from (A), *p*-values were calculated using Student's *t* test. Error bars represent SEM. (C) Quantification of maxillary nerves with GFP^+^ axon fibers from (A). (D) Single slice confocal images; Draper antibody stains in adult brains of control (*repo-gal4*/+) and *stat92e* ^RNAi^ (*stat92e* ^RNAi^/+;*repo-gal4*/+) backgrounds. Uninjured, one day after maxillary palp injury and one day after antennal ablation are shown. Dotted circles and boxes represent regions used for quantification of Draper immunoreactivity. (E) Quantification of data from (D), *p*-values were calculated using one-way ANOVA followed by Tukey's post hoc test, *n.s.*, not significant. Error bars represent SEM. (F) Western blot analysis of Draper protein levels in ∼3 adult central brain regions of control (*repo-gal4*/+), *stat92e^RNAi^* (*UAS-stat92e^RNAi^/+; repo-gal4/+*), and *draper^RNAi^* (*draper^RNAi^*/+;*repo-gal4/+*) genotypes. α-Tubulin was used as a loading control. Full blot located in Figure S3. (G) Quantification of Draper protein levels shown on Western blot after normalization to α-Tubulin, *n* = 3, *p*-values were calculated using one-way ANOVA followed by Tukey's post hoc test. (H) Real-time PCR analysis of *draper* mRNA levels in adult central brain region of control (*repo-gal4/+), drpr^RNAi^* (*draper^RNAi^*/+;*repo-gal4/+), pi3k92e^CAAX^(UAS-pi3k92e^CAAX^/+;repo-gal4/+), pi3k92e^RNAi^(UAS-pi3k92e^RNAi^/+;repo-gal4/+), and stat92e^RNAi^* (*stat92e^RNAi^*/+;*repo-gal4/+) genotypes*. Draper Ct values were normalized to ribosomal protein L32 and results are presented as fold induction relative to control. Values represent mean ± SEM from three independent RNA isolations. *p*-Values were calculated using one-way ANOVA and Dunnett's post hoc test.

We next sought to determine whether Stat92E was required for modulating glial responses to axonal injury and clearance of degenerating axonal debris. Stat92E function was knocked down specifically in glia by expressing a *UAS-stat92e^RNAi^* construct with the pan-glial *repo-Gal4* driver. In controls the majority of GFP^+^ maxillary palp ORN axon material was cleared from the brain 5 days after injury (Figure 3A–3C; Data S6). However, in *stat92e* ^RNAi^ animals, GFP^+^ axonal debris persisted 5 days after injury (Figure 3A–3C; Data S6). In contrast to depletion of the PI3K signaling cascade, glial *stat92e^RNAi^* suppressed clearance of antennal ORN axons even 15 days after injury (Figure S3A), indicating an essential requirement for STAT92E in glial clearance of axonal debris. We were able to confirm the *UAS-Stat92E* ^RNAi^ line efficiently targets *stat92e* as glial co-expression of a GFP-tagged stat92e molecule with the *stat92e^RNAi^* construct eliminated all Stat92E-GFP expression compared to controls (Figure S3B). These observations argue that Stat92E is an important regulator of glial engulfment activity in the adult brain.

On the basis of our identification of a Stat92E-dependent injury-responsive element in the *draper* gene, we predicted Stat92E would modulate glial phagocytic activity by regulating *draper* expression after axotomy. Draper is normally expressed in ensheathing and cortex glia throughout the uninjured brain and is up-regulated around the antennal lobe after antennal ORN axotomy (Figure 3D). Glial-specific knockdown of Stat92E led to nearly undetectable levels of Draper expression in the adult brain even prior to injury (Figure 3D and 3E; Data S7). We confirmed this widespread loss of Draper by performing Western blots on dissected adult brains from control, *stat92e^RNAi^*, and *draper^RNAi^* animals (Figures 3F, 3G, and S3; Data S8). In contrast to loss of PI3K signaling, knockdown of Stat92E in glia was also sufficient to potently suppress glial activation of *draper* after axotomy: *stat92e^RNAi^* animals exhibited no detectable increase in Draper levels after antennal ablation compared to controls. In addition, while Draper was localized specifically to severed ORN axons after maxillary palp ablation in controls, we found no detectable Draper localization to severed axons in *stat92e^RNAi^* animals (Figure 3D and 3E; Data S7). These data suggest that Stat92E is required to establish normal basal levels of Draper expression in glia, and dynamically regulates increased Draper levels as glia respond to axotomy.

STAT signaling is involved in multiple cellular processes including cell survival, differentiation, motility, and immunity [33]–[42]. To exclude the possibility that the defects we observed in *stat92e^RNAi^* animals were the result of abnormalities in glial cell development we used the conditional Gal80^ts^ system to specifically activate *stat92e^RNAi^* at adult stages. When temperature sensitive *stat92e^RNAi^* animals were raised and tested at 18°C, we found that glia efficiently cleared axonal debris and expressed normal levels of Draper (Figure S4; Data S9). However, when they were shifted to and tested at the restrictive temperature during adult stages (thereby activating the RNAi construct only after development was complete), we found that *stat92e^RNAi^* animals exhibited reduced expression of Draper and failed to clear degenerating axons (Figure S4; Data S9). Glial cell morphology (visualized with membrane-tethered GFP) and numbers (counted with α-Repo antibody nuclear staining) appear grossly normal in these animals, arguing that these phenotypes are not the result of glial cell loss in *stat92e^RNAi^* backgrounds (Figure S5A and S5B). Moreover, we found that adult-specific activation of the RNAi was reversible, as shifting these animals back to 18°C (thereby turning the RNAi off) re-established normal levels of Draper and initiated clearance of axonal debris (Figure S4; Data S9). Thus Stat92E functions in adult brain glia, where it modulates Draper expression and glial phagocytosis of degenerating axons.

Draper and the PTB domain-containing protein dCed-6 are both required for glial engulfment of degenerating axons and are expressed in glial cells in the adult brain [23]. To determine whether Stat92E broadly regulates engulfment gene expression we assayed dCed-6 levels in the adult brain in animals expressing *stat92e^RNAi^* in glia and found that dCed-6 was still present at high levels throughout the brain (Figure S5C and S5D). Interestingly, while dCed-6 levels appeared grossly normal in a *stat92e^RNAi^* background, dCed-6 was not recruited to severed maxillary palp axons 1 day after axotomy (Figure S5C). Thus, while Stat92E is necessary for dCed-6 recruitment to severed axons (i.e., glial responses to injury), basal levels of dCed-6 do not appear to be regulated by a Stat92E dependent mechanism.

Finally, we sought to determine whether Draper levels were regulated transcriptionally by STAT92E and/or PI3K signaling. We performed quantitative real-time PCR to measure *draper* transcript levels in dissected brains from control animals and animals expressing glial *draper^RNAi^*, *pi3k92e^CAAX^* (gain-of-function), *pi3k92e^RNAi^*, or *stat92e^RNAi^*. Consistent with STAT92E regulating *draper* at the transcriptional level, we found that glial RNAi for *stat92e* severely reduced *draper* transcripts to a level comparable to depletion with *draper^RNAi^* (Figure 3H; Data S10). In contrast, neither loss- or gain-of-function manipulation of PI3K92E resulted in a statistically significant difference in *draper* transcript levels. While this argues for a post-transcriptional mechanism of Draper regulation by PI3K signaling, we cannot rule out the possibility that PI3K at least partially regulates *draper* at the transcriptional level considering loss of PI3K resulted in a trend of decreased (∼40%) *draper* mRNA (Figure 3H; Data S10).

### Stat92E Transcriptional Activity Is Transiently Up-regulated in Glia after Axonal Injury

**Figure 4:**
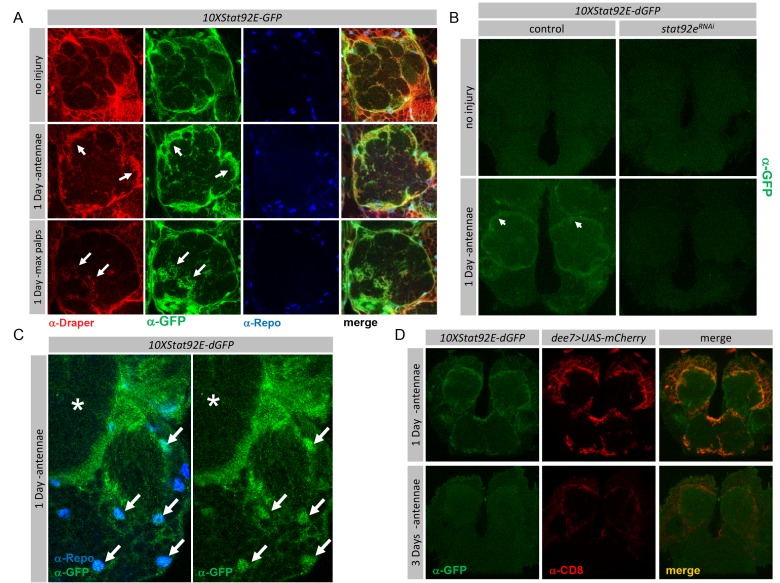
Stat92E transcriptional reporters are expressed in glia and transiently activated after axotomy. (A–D) Single slice confocal images; (A) 10XStat92E-GFP reporter expression in adult brain in uninjured control animals, one day after maxillary palp, and one day after antennal ablation. Glial nuclei are labeled with α-Repo (blue), glial membranes with α-Draper (red), and Stat92E transcriptional reporter activity with α-GFP (green). (B) 10XStat92E-dGFP in control (10xStat92E-dGFP/+;*repo-gal4*/+), and *stat92E^RNAi^* (10xStat92E-dGFP/*stat92e* ^RNAi^;*repo-gal4*/+) backgrounds, uninjured and one day after antennal ablation. (C) High magnification image showing colocalization of Repo^+^ glial nuclei (arrows) and 10XStat92E-dGFP reporter activity in cortex glial cells following injury. Asterisks mark antennal lobe region. (D) Colocalization of mCherry driven by *dee7-Gal4* with the 10xStat92E-dGFP reporter activity one and three days after antennal ablation.

To explore the dynamics of Stat92E signaling in adult brain glia we examined the expression patterns of transcriptional reporters for Stat92E activity [43]. These reporters have been previously shown to accurately reflect Stat92E transcriptional activity during development as well as in the adult [43]–[46]. We first used the *10XStat92E-GFP*, which harbors ten Stat92E binding sites driving expression of enhanced GFP. In co-stains with α-Draper and α-Repo (a glial nuclear marker) we observed quite specific glial expression of the Stat92E reporter in uninjured controls (Figure 4A). Moreover, after ablation of antennae we found strong GFP labeling of antennal lobe glia, and the GFP signal completely overlapped with Draper (Figure 4A). After ablation of maxillary palps we found GFP signals co-localized with Draper in glomeruli housing severed axons (Figure 4A). These data argue that Stat92E is active at a transcriptional level in adult brain glia.

The GFP driven by the *10XStat92E-GFP* reporter is quite stable and can perdure in cells for ∼20 hours after activation, which precludes our use of this construct to examine dynamic changes in Stat92E transcriptional activity. We therefore used a second reporter, *10XStat92E-dGFP*, which drives the expression of a rapidly degraded, destabilized GFP (dGFP), thereby allowing for increased temporal resolution of Stat92E activity. Prior to injury, we were unable to detect any activation of this Stat92E transcriptional reporter in adult brains (Figure 4B). However, beginning ∼16 hours after antennal ablation we detected *10XStat92E-dGFP* expression in cells surrounding the antennal lobe (Figure 4B, arrows). GFP intensity peaked at ∼24 hours after antennal ablation and disappeared by 48 hours after axotomy (Figure S6).

To confirm that activation of the *10XStat92E-dGFP* reporter after axotomy is Stat92E-dependent and glial specific, we knocked down Stat92e specifically in glia, severed axons, and assayed *10XStat92E-dGFP* activity. We found that glial-specific knockdown of Stat92E completely suppressed the axotomy-induced activation of the *10XStat92E-dGFP* transcriptional reporter (Figure 4B). Consistent with our observations of widespread activation of the *dee7-Gal4* driver in glia throughout the brain after injury, we also found that axonal injury led to broad activation of the *10XStat92E-dGFP* reporter in glial cells (Figure 4C) and expression of these two reporters colocalize in glial cells after axotomy (Figure 4D), indicating they are active in the same cells. Together these data indicate that Stat92E can transiently increase the transcriptional activation of target genes in glial cells throughout the brain after axonal injury, with the strongest increases in target gene activation occurring adjacent to injury sites.

### Stat92E-Dependent Activation of Draper Does Not Require Components of the Canonical JAK/STAT Signaling Pathway

STAT activity is generally regulated by the JAK signaling platform, and this pathway is conserved in all higher metazoans. The *Drosophila* JAK/STAT signaling pathway consists of a single JAK molecule, Hopscotch (hop) [47], and the cytokine like receptor Domeless (Dome) [48]. To determine if Stat92E-dependent activation of *draper* is mediated through canonical JAK/STAT signaling we drove RNAi constructs targeted against *hop*, and a dominant negative Domeless molecule, Domeless^ΔCYT^ [49], in glial cells, and assayed Draper expression and clearance of severed axons. Surprisingly, in each of these backgrounds we found Draper levels were similar to control animals and axons were efficiently cleared 5 days after injury (Figure S7A and S7B; Data S11). Reciprocally, we found a gain-of-function allele of *hop*, *hop^TUM^*, which has been shown in numerous assays to activate Stat92E transcriptional activity [43],[50],[51], failed to activate the*10XStat92E-dGFP* reporter in adult brain glia (Figure S7C). Notably, expression of an activated version of Stat92E, Stat92E^ΔNΔC^ [52], led to strong activation of the *10XStat92E-dGFP* reporter (Figure S7C). Stat92E^ΔNΔC^ has previously been shown to require phosphorylation at Y711 for activation [52]. We therefore expressed a version of Stat92E^ΔNΔC^ with a Y711F mutation in glia, and found it was insufficient for activation of the *10XStat92E-dGFP* reporter (Figure S7C). Together these data argue that while phosphorylated Stat92E mediates activation of Stat92E reporters in glia, canonical JAK/STAT signaling is neither necessary nor sufficient to activate Stat92E-dependent glial responses to axon injury.

### Signaling through the Draper Receptor Activates Stat92E-Dependent Transcriptional Up-regulation of draper

**Figure 5:**
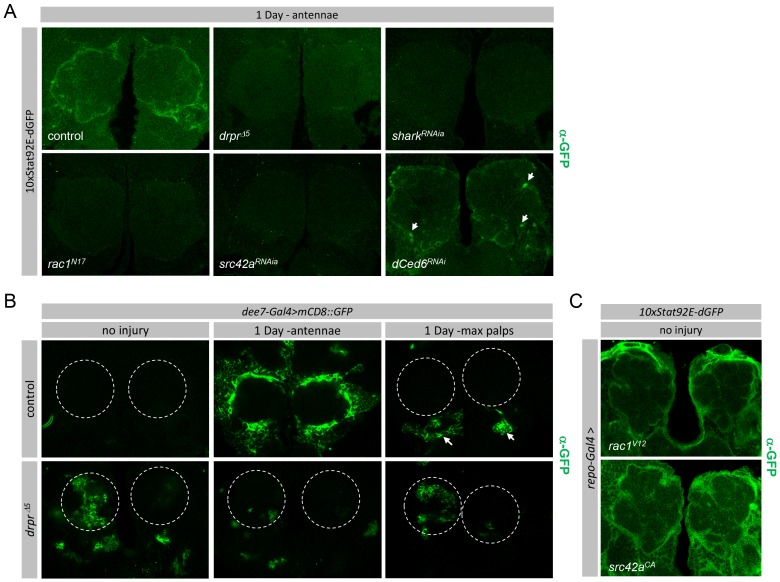
Injury-induced activation of Stat92E is downstream of the Draper signaling cascade. (A–C) Single slice confocal images; (A) Glial-specific knockdown of components of the Draper pathway in a *10XStat92E-dGFP* reporter background one day after antennal ORN axotomy; control (*10XStat92E-dGFP/+*) *draper* ^Δ*5*Δ*5*^ (*10XStat92E-dGFP/+, draper* ^Δ*5*^ */draper* ^Δ*5*^), *shark^RNAi^* (*shark^RNAi^*/*10XStat92E-dGFP; repo-Gal4*/+), *rac1^N17^*(*10XStat92E-dGFP/+; repo-Gal4*/*rac1^N17^*), *src42a^RNAi^* (*src42a^RNAi^*/*10XStat92E-dGFP; repo-Gal4*/+), and *dCed6^RNAi^* (*dCed6^RNAi^*/*10XStat92E-dGFP; repo-Gal4*/+) genotypes. (B) *dee7-Gal4* activity in control (*dee7-Gal4,UAS-mCD8::GFP*/+) and *draper* ^Δ*5/*Δ*5*^ (*dee7-Gal4,UAS-mCD8::GFP*/+, *draper* ^Δ*5*^ */draper* ^Δ*5*^) backgrounds. Uninjured, one day after antennal injury, and one day after maxillary palp injury are shown. Dotted circles indicate antennal lobes. (C) Glial-specific activation of components of the *draper* pathway in a *10XStat92E-dGFP* reporter background; *rac1^V12^* (*10XStat92E-dGFP/+; repo-Gal4*/*rac1^v12^*), and *src42a^ca^* (*10XStat92E-dGFP/+; repo-Gal4*/*src42a^ca^*).

To date Domeless is the only receptor known to positively regulate Stat92E signaling under normal physiological conditions [48],[53],[54]. Considering we were unable to demonstrate a role for Domeless signaling in activation of Draper after injury, we sought to determine whether Draper itself might have a role in modulating glial gene expression. We assayed *10XStat92E-dGFP* activation after axonal injury in *draper* ^Δ*5*^ null mutants and, intriguingly, loss of Draper resulted in a complete lack of *10XStat92E-dGFP* activation after axotomy (Figure 5A). Consistent with a direct requirement for Draper signaling in STAT92E-dependent activation of *draper* after axotomy, we also found a lack of activation of the *dee7-Gal4* reporter after axonal injury in *draper* ^Δ*5*^ animals (Figure 5B). These data demonstrate a direct role for the Draper receptor in modulating Stat92E-dependent changes in glial gene expression following local axonal injury.

We next explored whether other identified components of the Draper signaling pathway modulate Stat92E transcriptional activity after axon injury. Draper is thought to be phosphorylated by Src42a upon activation, initiating binding of the non-receptor tyrosine kinase Shark, which together, with Rac1 and dCed-6, promote engulfment [23],[25]. Interestingly, we found that glial-specific knockdown of Shark, Src42a, or Rac1 blocked injury-induced activation of the*10XSTAT92E-dGFP* transcriptional reporter (Figures 5A and S8A). This finding was confirmed using multiple RNAi lines and/or dominant negative alleles for each gene in the pathway (Figure S8A). However, while RNAi mediated knockdown of dCed-6 efficiently eliminated dCed-6 immunoreactivity (Figure S8B), axotomy-induced activation of *10XSTAT92E-dGFP* was still detectable in *dCed-6^RNAi^* animals (Figure 5A). Thus Draper, Src42a, Shark, and Rac1, but not dCed-6, are essential for Stat92E-dependent activation of transcriptional targets in glia responding to axonal injury. These are the first data that demonstrate a functional divergence between dCed-6 and Draper/Src42a/Shark function during engulfment signaling.

Since Src42a is known to signal downstream of Draper and has been shown to mediate Stat92E signaling in multiple contexts [25],[55],[56], we sought to determine whether Src42a activity was sufficient to activate Stat92E transcriptional reporters. Indeed, expression of a constitutively active Src42a molecule (Src42a^CA^) resulted in robust *10XStat92E-dGFP* reporter activation throughout brain (Figure 5C, refer to Figure 4B for control). Based on our previous genetic studies it appears that Src42a acts through modulating Rac1 activity. We therefore drove glial expression of a constitutively active Rac1 molecule (Rac1^v12^) and found this also robustly activated the *10XStat92E-dGFP* reporter throughout the adult brain (Figure 5C, refer to Figure 4B for control). These data are consistent with the notion that Src42a acts in glia downstream of Draper to activate Stat92E signaling through Rac1 after axonal injury.

In summary, our data suggest that axonal injury leads to Stat92E-dependent transcriptional up-regulation of *draper* through a Draper/Src42a/Shark/Rac1-dependent signaling cascade. While activation of Stat92E, Src42A, or Rac1 led to robust activation of the *10XStat92E* reporter, none were sufficient to increase basal Draper levels (unpublished data), suggesting additional transcriptional inputs are required for injury-induced transcriptional activation of draper.

### Draper Is a Primary Target for Stat92E during Glial Clearance of Degenerating Axons

**Figure 6:**
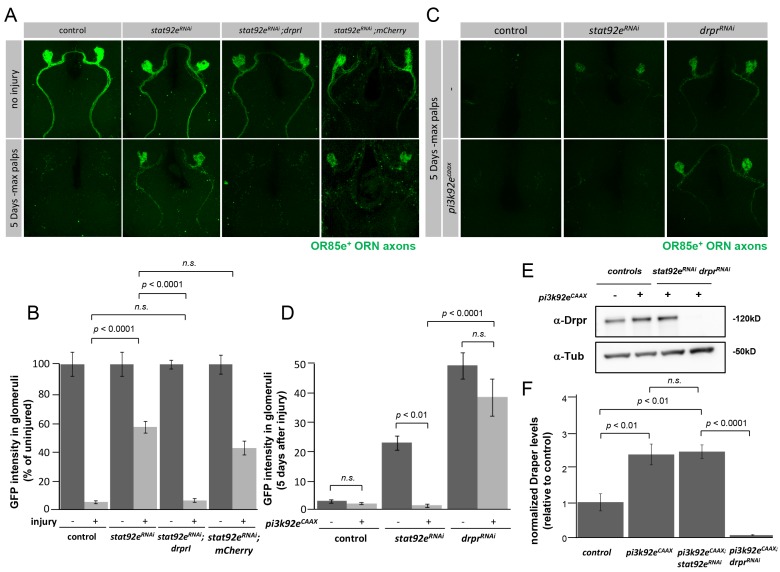
The *draper* gene is a primary target of Stat92E after axonal injury. (A) Z-stack confocal images; OR85e^+^ axons were labeled with GFP in control (*OR85e-GFP/+; repo-gal4/+*), *stat92e^RNAi^* (*stat92e^RNAi^*/*OR85e-GFP;repo-Gal4*/+), *stat92e^RNAi^/UAS-drprI (stat92e^RNAi^*/*OR85e-GFP;repo-Gal4*/*UAS-drpr-I)*, and *stat92e^RNAi^/UAS-mCherry (stat92e^RNAi^*/*OR85e-GFP;repo-Gal4*/*UAS-mCherry)* backgrounds. Uninjured and 5 days after maxillary palp ablation are shown. (B) Quantification of GFP intensities in 85e^+^ glomeruli from (A), *p*-values were calculated using one-way ANOVA followed by Tukey's post hoc, *n.s.*, not significant. Error bars represent SEM. (C) Z-stack confocal images of OR85e^+^ axons labeled with GFP; controls (OR85e-*GFP,Gal80^ts^*/+; *repo-gal4*/+; and *UAS*-pi3k92e^CAAX^/+;OR85e-*GFP,Gal80^ts^*/+; *repo-gal4*/+); *stat92e^RNAi^* (OR85e-*GFP,Gal80^ts^*/UAS-*stat92e^RNAi^*; *repo-gal4*/+; and *UAS-pi3k92e^CAAX^/+;OR85e-GFP,Gal80^ts^/UAS-stat92e^RNAi^;repo-gal4/+)*; and *drpr^RNAi^* (*OR85e-GFP,Gal80^ts^/UAS- drpr^RNAi^; repo-gal4/+*; and *UAS-pi3k92e^CAAX^/+, OR85e-GFP,Gal80^ts^/UAS-drpr^RNAi^;repo-gal4/+)*. Five days after maxillary palp ablation is shown. (D) Quantification of GFP intensities in 85e^+^ glomeruli from (C), *p*-values were calculated using one-way ANOVA and Tukey's post hoc analysis, *n.s.*, not significant. Error bars represent SEM. (E) Western blot analysis of Draper protein levels in ∼3 adult central brain regions of controls (OR85e-*GFP,Gal80^ts^*/+; *repo-gal4*/+; and *UAS*-pi3k92e^CAAX^/+;OR85e-*GFP,Gal80^ts^*/+; *repo-gal4*/+), *stat92e^RNAi^*, (*UAS-pi3k92e^CAAX^/+; OR85e-GFP,Gal80^ts^/UAS-stat92e^RNAi^;repo-gal4/+*), and *drpr^RNAi^* (*UAS-pi3k92e^CAAX^/+;OR85e-GFP,Gal80^ts^/UAS-drpr^RNAi^;repo-gal4/+*) backgrounds. α-Tubulin was used as a loading control. Full blot located in Figure S9. (F) Quantification of Draper protein levels shown on Western blot after normalization to α-Tubulin, *n* = 4, *p*-values were calculated using one-way ANOVA and Tukey's post hoc analysis.

Together our data argue that *draper* transcription is regulated by Stat92E after axotomy. Stat92E may regulate many genes after axotomy, or only a few critical targets essential for engulfment. To further explore the relationship between Stat92E and the *draper* gene, we over-expressed Draper in a *stat92e* ^RNAi^ background to determine whether resupplying Draper was sufficient to overcome the engulfment deficit observed in Stat92E knockdown animals. Remarkably, over-expression of Draper in *stat92e* ^RNAi^ animals led to a near complete rescue of the engulfment defect (Figure 6A and 6B; Data S12). As an alternate method to increase Draper levels we also drove glial expression of the activated PI3K molecule, PI3K92e^CAAX^, in the presence of *stat92e* ^RNAi^ or control *draper^RNAi^* animals. We found that activation of PI3K signaling was sufficient to increase Draper levels (Figures 6E, 6F, and S9; Data S13) and rescue engulfment defects in *stat92E^RNAi^* backgrounds (Figure 6C and 6D; Data S14).

Consistent with the ability of PI3K signaling to drive Draper expression independently of Stat92E, we also found that activated PI3K signaling was not sufficient to activate expression of the *10XStat92edGFP* reporter (Figures 6E, 6F, and S9). However, PI3K signaling appeared to sensitize brain glia to injury as glial PI3K92e^CAAX^ enhanced *10XStat92E-dGFP* activation after antennal ablation compared to controls (Figure S9), perhaps through driving increased Draper levels. From these data we conclude that *draper* is a critical target of Stat92E during glial responses to axonal injury. In addition, our data argue that activated PI3K signaling results in Stat92E-independent increases in Draper levels.

## Discussion

### PI3K Signaling and Stat92E Converge to Regulate Draper Expression in the Adult Brain

Our work identifies two new signaling pathways important for regulating glial engulfment function *in vivo*. First, we show that the PI3K signaling pathway modulates Draper levels in the healthy, uninjured brain as reduced PI3K signaling leads to dramatically decreased glial Draper and constitutive activation of PI3K signaling leads to Draper up-regulation. However, depletion of PI3K signaling components delays but does not completely block the ability of glial cells to up-regulate Draper levels or clear axonal debris in response to axotomy, perhaps due to positive signaling through the small amount of Draper that remains under these conditions. Second, we identify Stat92E as a potent regulator of both basal and injury induced Draper levels in adult brain glia. Loss of Stat92E in mature glia results in significantly decreased *draper* transcript levels and a near complete loss of Draper protein in the uninjured brain.

What is the relationship between PI3K signaling and Stat92E in regulating basal levels of Draper in the healthy brain? On the basis of our analysis of *draper* mRNA levels we speculate that STAT92E regulates *draper* at least in part at the transcriptional level. It appears unlikely that Stat92E functions downstream of PI3K signaling since loss of Stat92E in a constitutively activated PI3K background did not suppress PI3K-dependent increases in Draper levels, and gain-of-function PI3K was sufficient to rescue reduced Draper levels and engulfment defects in *stat92e^RNAi^* animals. STAT molecules have been shown to be capable of acting as adaptor molecules for receptors that ultimately lead to activation of PI3K signaling [57]–[59], therefore Stat92E could function in adult brain glia upstream of PI3K signaling in a non-transcriptional manner to regulate basal levels of Draper. Finally, Stat92E might transcriptionally regulate key molecules required for activation or execution of PI3K signaling. In such a situation Stat92E and PI3K signaling could modulate Draper levels through parallel mechanisms, but both would be required for expression of appropriate levels of Draper in the adult brain.

A role for PI3K signaling in phagocytic function appears to be conserved from *Drosophila* to mammals. Activation of PI3K signaling occurs downstream of the Fcγ receptor [60]–[63]. This finding is intriguing in light of the fact that Draper appears to act as an ancient immunoreceptor, activating a Src family kinase signaling cascade through ITAM/ITIM-dependent mechanisms [25],[27]. PI3K signaling is required for efficient formation of the phagocytic cup in macrophages [61],[64],[65], and loss of PI3K has been reported to lead to a delay in clearance of cell corpses and myelin [62],[66],[67]. This latter finding argues for conservation of a requirement for PI3K signaling even among glial cell types in *Drosophila* and mammals. In our study we also find a delay of axonal clearance when PI3K signaling is suppressed, but ultimately axons are cleared. We speculate this phenotype is a result of decreased expression of Draper since we can fully rescue loss of PI3K signaling phenotypes by resupplying Draper. This observation argues strongly that a key role for PI3K signaling in phagocytic function is the regulation of engulfment factors, and in particular Draper.

### Axonal Injury Causes Widespread Activation of Glial Cells throughout the Drosophila Brain

Ablation of *Drosophila* third antennal segments leads to axotomy of all antennal ORNs and Wallerian degeneration of ∼1,200 ORN axons and their synapses within the antennal lobe of the brain. We previously observed a robust increase in Draper levels in glia surrounding the antennal lobe after antennal injury (but not elsewhere) and proposed that Draper increases were only local [23],[24]. Using the *dee7-Gal4* and *10XStat92E-dGFP* reporters we have now shown that severe axonal injury (i.e., axotomy of nearly all ORNs) in the antennal lobe is sufficient to induce a transcriptional response in glial cells throughout the entire *Drosophila* brain (Figures 2B–2D, 4B–4D, 5A, and 5B). How can glial cells in distant parts of the brain receive signals that an injury has occurred? We can envision at least two scenarios to explain this observation. First, severed axons could release signals that act at a long distance to activate glia. If so, the reception of this axon-derived signal by glia would be Draper-dependent, since we show that Draper signaling is required for activation of both the *dee7-Gal4* and *10XStat92E-dGFP* reporters in glia after injury. Alternatively, spreading of an injury signal throughout the brain could be accomplished by glial to glial signaling. Astrocytes are indeed heavily coupled in mammals [68] and the same could be true in *Drosophila* adult brain glia where signals could spread through gap junction-dependent mechanisms. In the future it will be very exciting to define the molecules that regulate the spreading of the injury signal to distant glial subtypes, and determine their functional role in brain recovery from trauma.

**Figure 7:**
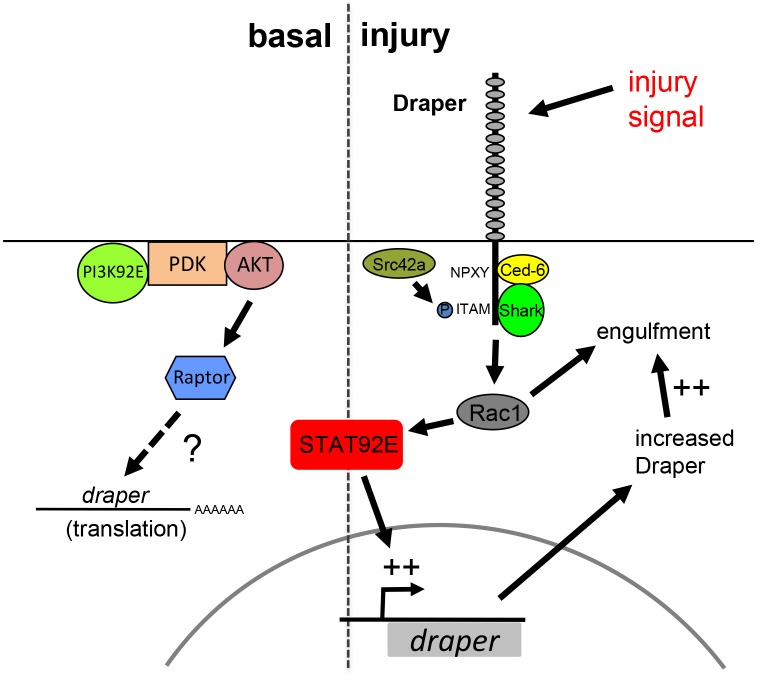
Basal and injury-induced Draper expression are regulated through two distinct pathways. Basal levels of Draper expression are dependent upon the convergent activities of PI3K and Stat92E signaling. After injury Draper signals through the Src family kinase signaling cascade (Src and Shark), and Rac1, and transcriptionally activates its own expression in a Stat92e-dependent fashion. Increased levels of Draper (and perhaps other engulfment genes) likely enhance the ability of glia to engulf axonal debris.

In mammals, it is widely accepted that reactive glial responses are graded according to the severity of the brain injury. Here we show that *Drosophila* glia also respond in a graded way to ORN injury: axotomy of a small number of ORN axons by maxillary palp ablation led to *dee7-Gal4* activity in a small subset of cells, while severing the majority of ORNs (∼85%) by antennal ablation led to a more dramatic increase in the activation of two separate reporters. We propose that relatively mild injuries promote signaling through the Draper pathway in a limited number of cells close to the site of injury while more severe injuries result in widespread activation of the Draper pathway in cells throughout the entire brain, even those distant from the injury site. Presumably up-regulation of engulfment factors enhances the ability of glia to clear neuronal debris (Figure 7). Such a mechanism whereby glial transcriptional responses are activated downstream of the very pathways that drive glial phagocytic activity would allow glia to directly modulate their engulfment capacity. Since it is likely that Draper ligands are present on engulfment targets, transcriptional activation of glial engulfment genes would ultimately be regulated by extracellular levels of “eat me” cues on degenerating axons.

### A Draper→Stat92E←draper Gene Auto-Regulatory Loop Increases Glial Engulfment Activity after Axotomy

We propose a novel injury-induced auto-regulatory loop whereby activation of Draper in glial cells responding to axonal injury leads to downstream signaling through Src42a/Shark/Rac1, and in turn Stat92E-dependent transcriptional activation of the *draper* locus. We provide multiple lines of evidence that activation of glial cells after local axotomy involves Stat92E-dependent transcriptional activation of *draper*: RNAi knockdown of Stat92E leads to a loss of injury-induced Draper expression, mutation of a single Stat92E binding site in the *dee7* enhancer element leads to ∼40%–50% reduction in *dee7-Gal4* activity upon injury, the *10XStat92E-dGFP* transcriptional reporter is activated in glial cells upon injury, and activation of both *10XStat92E-dGFP* and the *dee7-Gal4* reporter are lost in *draper* null animals. We provide compelling evidence that the *dee7* reporter activity resembles injury induced transcriptional activation of Draper: it expresses in the subtype of glia known to respond to injury and only after axotomy; it correlates with our data showing a larger increase in overall Draper up-regulation in the brain during the more severe antennal ablation; expression of the reporter is lost in *drpr* mutant animals; and its expression overlaps with the 10XStatDGFP reporter activity. As with all transcriptional reporters it remains to be determined whether it fully recapitulates endogenous *draper* regulation after injury, nevertheless, the *dee7-Gal4* element will be an extremely useful tool with which to dissect pathways mediating axon injury-induced molecular changes in glial transcription. Finally, based on the presence of STAT92E binding sites in the *draper* locus, and the requirement of these for maximal activation of the injury-responsive *dee7-Gal4* element, we suspect the *draper* gene may be a direct transcriptional target of STAT92E after axotomy.

Since mutation of the STAT92E binding sites in the *dee7* element only led to a partial inhibition of *dee7* activity after injury, it seems likely that Stat92E acts in cooperation with other factors. Evidence from mammals suggests that based on their weak transcriptional activation, STAT proteins often act in combination with other transcription factors for maximal target gene activation. Interestingly, we recently showed JNK signaling is required in glial cells for up-regulation of Draper after axotomy and that transcriptional reporters for JNK pathway signaling to dAP-1 increased in glia after axonal injury [69]. Mammalian STAT and AP-1 act together in reactive microglial cells to mediate the up-regulation of inflammatory proteins [70],[71]. It therefore remains an interesting possibility that combinations of Stat92E binding sites, AP-1 binding sites, and binding sites for other unidentified co-factors are involved in precisely modulating levels of Draper expression in the context of injury.

Our work has revealed a novel role for engulfment signaling pathways (i.e., Draper signaling) in the regulation of glial transcription after local axon injury, and revealed that Stat92E acts downstream. Despite the fact that Stat92E signaling is essential for a wide range of developmental processes in *Drosophila*, Draper is only the second receptor implicated in positively regulating the ability of Stat92E to activate transcriptional targets *in vivo* [48],[53],[54]. At the same time our work identifies the *draper* gene as a novel target for Stat92E—only three other genes, *eve*, *crb*, and *dome*, have been shown by *in vivo* analyses to be directly regulated by Stat92E [72]–[74]. Our work therefore enriches our understanding of the physiological roles for engulfment receptors and Stat92E signaling in the modulation of glial responsiveness to axonal injury.

We anticipate a similar role for mammalian engulfment receptors and STATs in reactive gliosis. Indeed, activation of STAT molecules in mammalian glial cells (determined primarily by using phospho-STAT-specific antibodies) has been widely reported in response to focal brain lesion [75],[76], traumatic brain injury [77],[78], and spinal cord injury (SCI) [79]. STAT3 is a critical modulator of reactive gliosis: loss of STAT3 from astrocytes in the context of SCI has been shown to lead to attenuated activation of GFAP expression, a lack of astrocyte hypertrophy, and reduced formation of the glial scar. This in turn results in increased demyelination, enhanced inflammation, and less efficient motor axon recovery [80],[81]. IL-6 has been shown to act upstream of STAT3-dependent GFAP induction in Schwann cells during peripheral nerve regeneration, but both the source of IL-6 *in vivo* and how STATs are activated in glia after injury remains unclear [82]. Based on this study we speculate mammalian STATs may be activated in reactive glia by the Draper orthologs MEGF10 or Jedi.

## Materials and Methods

### Fly Strains and Molecular Biology

The following fly strains were used: (1) *UAS-pdk1*, VDRC 18736, (2) *UAS-raptor^RNAi^*, VDRC 13112, (3) *UAS-pi3k92e^RNAi^*, VDRC 38986, (4) *UAS-pi3k92e^caax^*, Bloomington Stock 8294 (5) *UAS-mCD8::GFP* [83] (II), (6) *UAS-mCD8::GFP* [83] (III), (7) *OR85e*-*mCD8::GFP* [84], (8) *UAS-stat92e^RNAi^*, VDRC 43866, (9) *UAS-drpr^RNAi^* [24] (10) *repo-Gal4*, (11) *tubulin-Gal80^ts^*, (12) *repo-Gal4,UAS-mCD8::GFP*, (13) *10XStat92E-GFP* [43], (14) *10XStat92E-GFP* [43], (15) *UAS-rac1^V12^*, Bloomington Stock 6291, (16) *UAS-Src42a^CA^*, Bloomington Stock 6410, (17) *UAS-src42a^RNAi^* VDRC 26019 (18) *UAS-src42a^RNAi^* VDRC 100708, (19) *UAS-rac1^RNAi^* VDRC 49247, (20) *UAS-rac1^N17^*, Bloomington Stock 6292, (21) *UAS-shark^RNAi^* [25] (22) *UAS-shark^RNAi^*, VDRC 105706 (23) *UAS-Drpr-I* [27], (24) *drpr* ^Δ5^, [24], (25) UAS-mCherry, (26) and *yw*.

The *dee7-Gal4* construct was made by PCR amplification of the 2,619 bp fragment from the Draper BACR17K18 clone using the following primers: forward 5′caccagacctactcttagctctgatggagg-3′, reverse 5′-gtttgtgtttccatggattcaggcttggg-3′. The PCR product was purified using a Qiagen Gel Purification kit, directionally cloned into the Invitrogen pENTR/D-TOPO vector and transformed into One Shot Competent Cells using the pENTR/D-TOPO Cloning Kit (Invitrogen catalog number K2400-20). Colonies were prepped using the Qiagen miniprep kit. The *dee7* fragment was then shuttled into the pBGUw destination vector [15] using the Invitrogen Gateway LR Clonase Enzyme and transformed into heat shock competent DH5α cells. The construct was sequence verified and transgenic flies were generated by Best Gene Inc. using the PhiC31 targeted integration system.

To generate the *dee7^MUT^-GAL4* construct, the *dee7*/TOPO construct was used as a template and PCR was carried out using Invitrogen Quick Change II Site-Directed Mutagenesis kit (catalog number 200523) with the following primers (mutation sites underlined): forward-5′ CTG TGC CGA ACA CGT TAA CCA TTG AAA AAT CTC GC 3′, reverse-5′ GCG AGA TTT TTC AAT GGT TAA CGT GTT CGG CAC AG 3′. A DpnI digestion was performed and DNA was transformed into XL-1 Blue super competent cells and plated. Colonies were prepped using the Qiagen miniprep kit and the mutations were verified by sequencing (Genewiz). The *dee7^MUT^* enhancer fragment was then shuttled into the pBGUw vector using methods described above and transgenic flies were generated by Best Gene Inc. using PhiC3 targeted integration.

### Olfactory Neuron Injury Protocol, Immunohistochemistry, and Confocal Microscopy

Maxillary palp and third antennal segment ablations, adult brain dissections, and antibody stainings were performed using previously described methods [24],[85]. Samples were mounted in Vectashield (Vector Laboratories) antifade reagent and viewed on a Zeiss LSM5 Pascal confocal microscope. In all experiments, laser settings were kept identical for all brains imaged as part of that experiment. For Figure 2C, single slice confocal images of approximately the same depth in the brain were identified and total intensity of GFP in the brain was measured. The minimum threshold was set at 3 to eliminate most background and the maximum threshold was set at the maximum value of 255. Measures of GFP intensity within maxillary palp glomeruli were performed as previously described [24]. For Figure 3E, Draper expression after maxillary palp injury was measured from single confocal slices at the depth of the OR85e-innervated glomerulus. A circle was drawn around the area of the OR85e^+^ innervated glomerulus and total intensity of Draper was measured. Draper expression after antennal injury was measured from single confocal slices about half way through the antennal lobe. A fixed area rectangle at the edge of the antennal lobe was used to measure total intensity of Draper. All quantification of measurements was performed using Image J software. The following antibodies were used: 1∶200 mouse anti-GFP (Invitrogen), 1∶500 rabbit anti-Draper [86], 1∶500 rat anti-dCed6 [87], 1∶200 FITC anti-mouse IgG, 1∶200 Cy3 anti-rabbit IgG, 1∶200 Cy3 anti-rat IgG (Jackson ImmunoResearch). Flies were raised and maintained at 25°C unless otherwise noted. In experiments utilizing Gal80^ts^ flies were raised at 18°C and shifted to 30°C for at least 7 days prior to injury or dissection.

### Western Blots

*Drosophila* brains of the indicated genotype were dissected in PBS and homogenized in SDS loading buffer (60 mM Tris [pH 6.8], 10% glycerol, 2% SDS, 1% mercaptoethanol, 0.01% bromophenol blue). For Western analysis, samples containing approximately three brains were loaded onto 10% SDS-PAGE gels (BioRad), transferred to nitrocellulose membranes (BioRad), and probed with rabbit α-Draper antibody [86] at 1∶1,000 diluted in PBS/0.01% Tween-20/5% BSA. Blots were incubated overnight at 4°C, washed several times in PBS/0.01% Tween-20, and probed with the appropriate HRP conjugated secondary antibody for 2 hours at room temperature. Additional washes were performed and the blot was developed using chemiluminescence (Amersham ECL Plus), and detected with a Fujifilm Luminescent imager. The protein blot was stripped with mild stripping buffer (0.2M glycine, 0.1% sodium dodecyl sulfate, 1% Tween [pH 2.2]) at room temperature followed by washes in 1× PBS and 1× PBS+0.01% Tween-20 and then reprobed with mouse-tubulin (Sigma), 1∶1,000.

### Real-time PCR

*Drosophila* central brain regions were dissected in Jan's saline (1.8 mM Ca^2+^) and immediately frozen on dry ice. Total RNA was extracted in Trizol and the aqueous phase was passed over an Omega Bio-Tek E.Z.N.A MicroElute RNA Clean-Up Column with an on-column DNAase I treatment (Omega). RNA concentration was determined on a Nanodrop 2000c spectrometer (Thermo Scientific). RNA was diluted to equal concentration and 125 ng of total RNA was reverse-transcribed with the SuperScript VILO cDNA synthesis Kit for 2 h at 42°C.Relative quantification of gene expression was carried out on an ABI 7000 Real-Time PCR machine. The following Taqman assays (Applied Biosystems) were used: (i) Ribosomal protein L32 (ABI pre-made assay Dm02151827_g1) (ii) Draper-I custom assay, F-primer, TGTGATCATGGTTACGGAGGAC; R-primer, CAGCCGGGTGGGCAA; probe, CGCCTGCGATATAA [27]. Assay efficiencies were experimentally determined (RpL32, 102%; Draper-I, 103%; using a 5-point dilution series of cDNA spanning a 20-fold range in concentration. The raw threshold cycle (Ct) of the normalization control (RpL32) did not vary by more than 0.5 cycles across all time points analyzed. Statistical analysis (ANOVA, with Dunnett's Multiple Comparisons Test) was performed on 2^−ΔCt^ values. Draper Ct values were normalized to ribosomal protein L32 and results are presented as fold induction relative to uninjured levels.

## Supporting Information

Figure S1 Glial specific expression of a dominant negative PI3K92E results in decreased Draper protein levels and delayed axon clearance 5 days after injury. (A) Single slice confocal images of adult brains stained with Draper antibody and Z-stack confocal images of OR85e^+^ axons labeled with GFP in control (*OR85e-GFP,Gal80^ts^*/+; *repo-gal4*/+) and PI3K dominant negative backgrounds (*OR85e-GFP,Gal80^ts^*/+; *repo-gal4*/*UAS-pi3k92e^A2860C^*). Uninjured and 5 days after maxillary palp ablation are shown. (B) Quantification of GFP intensities in 85e^+^ glomeruli from (A), *p*-values were calculated using Student's *t* test, Error bars represent SEM. (C) Image of full Western blot for bands shown in Figure 1.
(TIF)

Figure S2 Simultaneous mutation of tandem Stat92e binding sites in the *dee7* enhancer element does not affect *dee7* activity after antennal ablation. (A) Schematic representation of the *draper* gene. Asterisks indicate location of the tandem Stat92E binding sites in the *dee7* enhancer region. Underlined letters indicate potential Stat92E binding motifs. Red letters show nucleotides mutated in the *dee7^MUT2^-Gal4* element. (B) Single slice confocal images of antennal lobe regions; *dee7-Gal4* or *dee7^MUT2^-Gal4* driving two copies of *UAS-mCD8::GFP (dee7>2XmCD8::GFP, dee7^MUT2^>2XmCD8::GFP)*. One day after antennal ablation and four days after antennal ablation are shown. (C) Quantification for (B), *p*-values were calculated using Student's *t* test, *n.s.*, not significant. Error bars represent SEM, *n*≥5 for all.
(TIF)

Figure S3 Glial specific knockdown of Stat92E inhibits axon clearance for at least 15 days after antennal ablation. (A) Z-stack confocal images of OR67b^+^ axons labeled with GFP in control (*OR67b-GFP/+;repo-gal4*/+) and *stat92e* ^RNAi^ (*OR67b-GFP/UAS-stat92e* ^RNAi^;*repo-gal4/+*) backgrounds. Uninjured and 15 days after antennal ablation are shown. (B) Single slice confocal images of adult brains stained with α-Draper and α-GFP in control (*UAS-stat92e-GFP/repo-gal4*), and *stat92e* ^RNAi^ (*UAS-stat92e* ^RNAi^/+; *UAS-stat92e-GFP*/*repo-gal4*) backgrounds. (C) Image of full Western blot for bands shown in Figure 3.
(TIF)

Figure S4 Stat92E is required in the adult brain for *draper* expression and glial engulfment function. (A) Z-stack confocal images of OR85e-GFP axons, single slice confocal images of Draper antibody stain; *UAS-stat92e* ^RNAi^ was driven in all glia using *repo-Gal4* in a background containing the temperature sensitive Gal4 repressor, Gal80^ts^. Axons (OR*85e-GFP*, green) and Draper levels (red) are shown in control (*OR85e-GFP,Gal80^ts^/+;repo-gal4/+*) or *stat92e^RNAi^* knockdown (*OR85e-GFP,Gal80^ts^/stat92e^RNAi^;repo-Gal4*/+) animals. Temperature shifts were performed as follows: 18°C indicates that flies were raised and kept at 18°C throughout experiment. 18°C–30°C indicates that flies were raised at 18°C and then shifted to 30°C as adults for 7 days prior to and 5 days following maxillary palp ablation. 18°C–30°C–18°C indicates that flies were raised at 18°C, shifted to 30°C as adults for 7 days, and then shifted back to 18°C for 7 days prior to and 5 days following maxillary palp ablation. (B) Quantification of GFP intensities in 85e^+^ glomeruli from (A). Error bars represent SEM, *n*≥10.
(TIF)

Figure S5 Glial specific knockdown of STAT92E does not disrupt glial morphology or result in loss of dCed-6 protein levels. (A) Single slice confocal images of adult brains of indicated genotype, (UAS-*stat92e^RNAi^/+;repo-Gal4,UAS-mCD8::GFP/Gal80^ts^*) one day after maxillary palp injury. Glial nuclei are labeled with anti-Repo (blue), glial membranes are labeled with GFP (green), and anti-Draper staining is shown (red). Flies were raised at 18°C and either kept at 18°C throughout the experiment or shifted to 29°C for 7 days prior to dissection. At the restrictive temperature of 18°C some repo-gal4 driven GFP is detectable but Draper staining looks grossly normal and glia are able to respond to injury one day after maxillary palp ablation. In flies shifted to the restrictive temperature, Draper staining is significantly reduced and no glial membranes are recruited to severed axons one day after maxillary palp injury. However, glial cell nuclei and membranes appear grossly normal in the stat92eRNAi flies. (B) Single slice confocal image of adult brain, high magnification view of cortex region of the brain. Asterisk indicates that while Draper is absent in the stat92e^RNAi^ animals, glial morphology is normal. (C) Adult brains one day after maxillary palp removal, stained with α-dCed6 or α-Draper in control (OR85e-GFP,Gal80ts/+;repo-Gal4/+), and *stat92e^RNAi^* (OR85e-GFP,Gal80ts/*stat92e^RNAi^*;repo-Gal4/+) backgrounds. (D) α-Draper Western blot performed in duplicate on ∼3 adult central brain regions per lane in control (OR85e-GFP,Gal80ts/+;repo-Gal4/+), and *stat92e^RNAi^* (OR85e-GFP,Gal80ts/*stat92e^RNAi^*;repo-Gal4/+) backgrounds. α-Tubulin was used as a loading control.
(TIF)

Figure S6 48 hour time course showing activation of the 10XStat92E-dGFP reporter following antennal ablation. (A) Single slice confocal images of adult brains; 10XStat92E-dGFP reporter activity in an uninjured brain and at various timepoints after injury, 12 hours, 16 hours, 24 hours, 36 hours, and 48 hours.
(TIF)

Figure S7 Glial activation of Stat92E activity after axotomy is not mediated by canonical JAK/STAT signaling. (A) *repo-gal4* was used to drive two independent UAS-*hop* ^RNAi^ constructs and a UAS dominant negative domeless allele; single slice confocal images of Draper antibody stain and Z-stack confocal images of OR85e-GFP axons in control (OR85e-GFP/*hop* ^RNAi(a)^;*repo-gal4*/+), hop^RNAi(a)^ (OR85e-GFP/+;*repo-gal4*/+) hop^RNAi(b)^ (OR85e-GFP/*UAS- hop* ^RNAi(b)^;*repo-gal4*/+), *domeless* ^ΔCYT^ (OR85e-GFP/*UAS-domeless* ^ΔCYT^;*repo-gal4*/+) (B) Quantification of data from (A). Error bars represent SEM, *n*≥10. (C) Single slice confocal images of indicated genotype in 10XSTATd-GFP background; control (*10XStat92E-dGFP*/+;*repo-gal4/+), hop^TUM^*(*hop^TUM^/X;10XSTAT92E-dGFP*/+;*repo-gal4/+*),*stat92e* ^ΔNΔC^ (*10XSTAT92E-dGFP*/+;*repo-gal4/UAS- stat92e* ^ΔNΔC^), *stat92e* ^ΔNΔCY711F^ (*10XStat92E-dGFP*/*stat92e* ^ΔNΔCY711F^;*repo-gal4/+*).
(TIF)

Figure S8 RNAi mediated knockdown of rac1, shark, and src42a results in loss of Stat92E transcriptional reporter activity after injury. (A) Glial-specific knockdown of components of the *draper* pathway in a *10XStat92E-dGFP* reporter background one day after antennal ORN axotomy; control (*10XStat92E-dGFP/+*), *shark^RNAi^* (*shark^RNAi^*/*10XStat92E-dGFP; repo-Gal4*/+), *rac1^RNAi^*(*rac1^RNAi^*/+;*10XStat92E-dGFP/+; repo-Gal4*/+) and *src42a^RNAi^* (*src42a^RNAi^*/*10XStat92E-dGFP; repo-Gal4*/+). (B) Single slice confocal images of adult brains stained with α-dCed6 in control, (OR85e-GFP,Gal80ts/+;repo-Gal4/+), and *d-ced6^RNAi^* (OR85e-GFP,Gal80ts/*d-ced6^RNAi^*;repo-Gal4/+) backgrounds.
(TIF)

Figure S9 Constitutively active PI3K signaling does not activate the 10XStat-dGFP reporter. (A) Single slice confocal images of the adult brains stained with α-Draper or α-GFP in control (*repo-Gal4/+; and 10XStat92E-dGFP/+;repo-Gal4/+*), and *pi3k92e^CAAX^* (*pi3k92e^CAAX^/+;repo-Gal4/+; and pi3k92e^CAAX^;10XStat92E-dGFP/+;repo-Gal4/+*) backgrounds. Uninjured, one day after maxillary palp injury and one day after antennal injury are shown. (B) Image of full Western blot for bands shown in Figure 6.
(TIF)

Data S1 Excel spreadsheet containing the raw numerical data and statistical analyses for Figure 1A and 1B.
(XLSX)

Data S2 Excel spreadsheet containing the raw numerical data and statistical analyses for Figure S1.
(XLSX)

Data S3 Excel spreadsheet containing the raw numerical data and statistical analyses for Figure 1D and 1E.
(XLSX)

Data S4 Excel spreadsheet containing the raw numerical data and statistical analyses for Figure 2B and 2C.
(XLSX)

Data S5 Excel spreadsheet containing the raw numerical data and statistical analyses for Figure S2B and S2C.
(XLSX)

Data S6 Excel spreadsheet containing the raw numerical data and statistical analyses for Figure 3A–3C.
(XLS)

Data S7 Excel spreadsheet containing the raw numerical data and statistical analyses for Figure 3D and 3E.
(XLS)

Data S8 Excel spreadsheet containing the raw numerical data and statistical analyses for Figure 3F and 3G.
(XLSX)

Data S9 Excel spreadsheet containing the raw numerical data and statistical analyses for Figure S4.
(XLS)

Data S10 Excel spreadsheet containing the raw numerical data and statistical analyses for Figure 3H.
(XLSX)

Data S11 Excel spreadsheet containing the raw numerical data and statistical analyses for Figure S7.
(XLS)

Data S12 Excel spreadsheet containing the raw numerical data and statistical analyses for Figure 6A and 6B.
(XLSX)

Data S13 Excel spreadsheet containing the raw numerical data and statistical analyses for Figure 6E and 6F.
(XLSX)

Data S14 Excel spreadsheet containing the raw numerical data and statistical analyses for Figure 6C and 6D.
(XLSX)

Methods S1 Supporting methods and references.
(DOC)

## Abbreviations

CNS: central nervous system
DEE: Draper enhancer element
ET-1: endothilin-1
GFP: green fluorescent protein
ORN: olfactory receptor neuron
PI3K: phosphoinositide 3-kinase
SEM: standard error of the mean
STAT: signal transducer and activator of transcription
