# Supplementary figures and images for: PI3K Signaling and Stat92E Converge to Modulate Glial Responsiveness to Axonal Injury
Source: PLoS Biol. 2014 Nov 4;12(11):e1001985. doi: 10.1371/journal.pbio.1001985 (PMC4219656; doi:10.1371/journal.pbio.1001985)

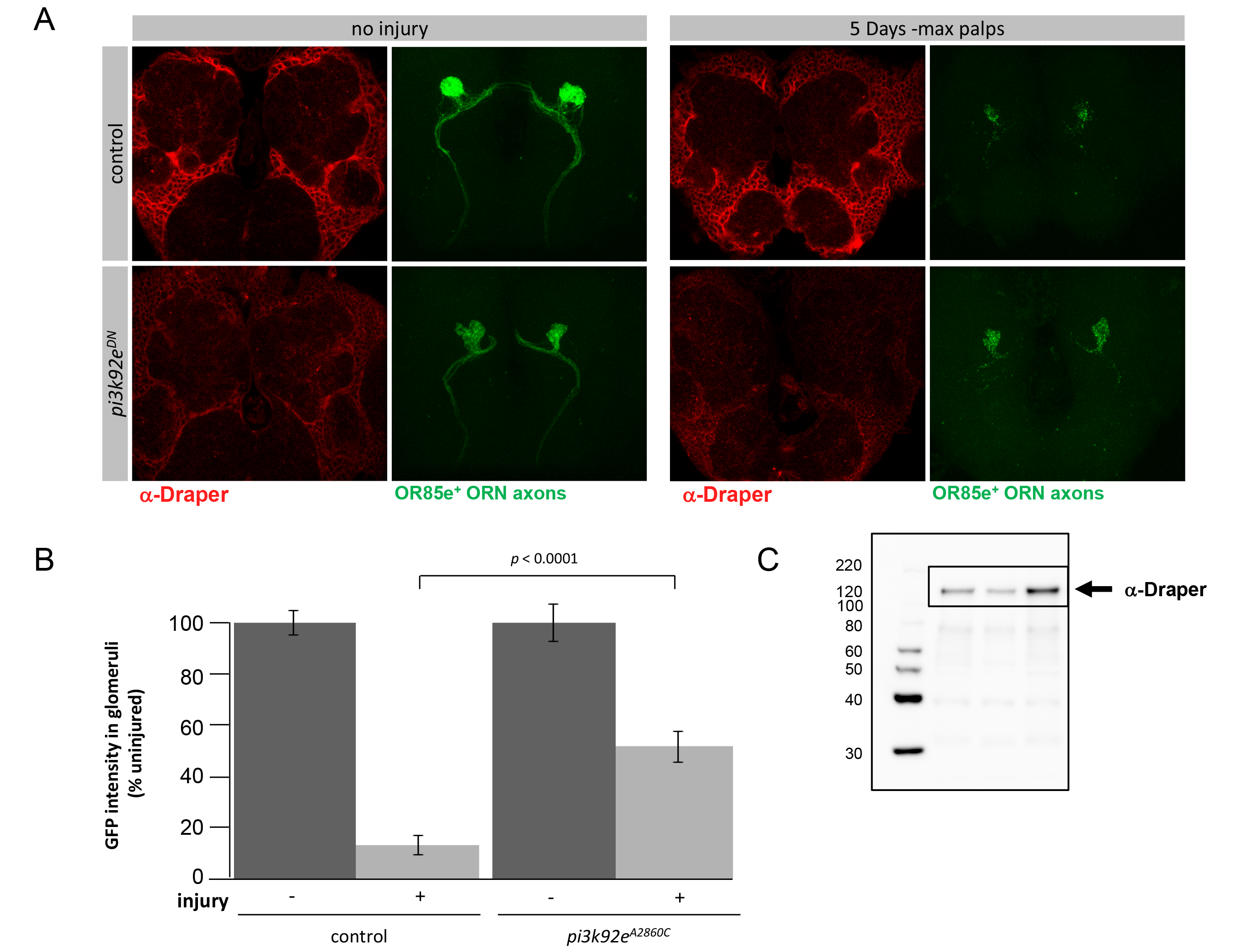

Supplement: Figure S1 — Glial specific expression of a dominant negative PI3K92E results in decreased Draper protein levels and delayed axon clearance 5 days after injury. (A) Single slice confocal images of adult brains stained with Draper antibody and Z-stack confocal images of OR85e+ axons labeled with GFP in control (OR85e-GFP,Gal80ts/+; repo-gal4/+) and PI3K dominant negative backgrounds (OR85e-GFP,Gal80ts/+; repo-gal4/UAS-pi3k92eA2860C). Uninjured and 5 days after maxillary palp ablation are shown. (B) Quantification of GFP intensities in 85e+ glomeruli from (A), p-values were calculated using Student's t test, Error bars represent SEM. (C) Image of full Western blot for bands shown in Figure 1. (TIF) [file pbio.1001985.s001.tif]

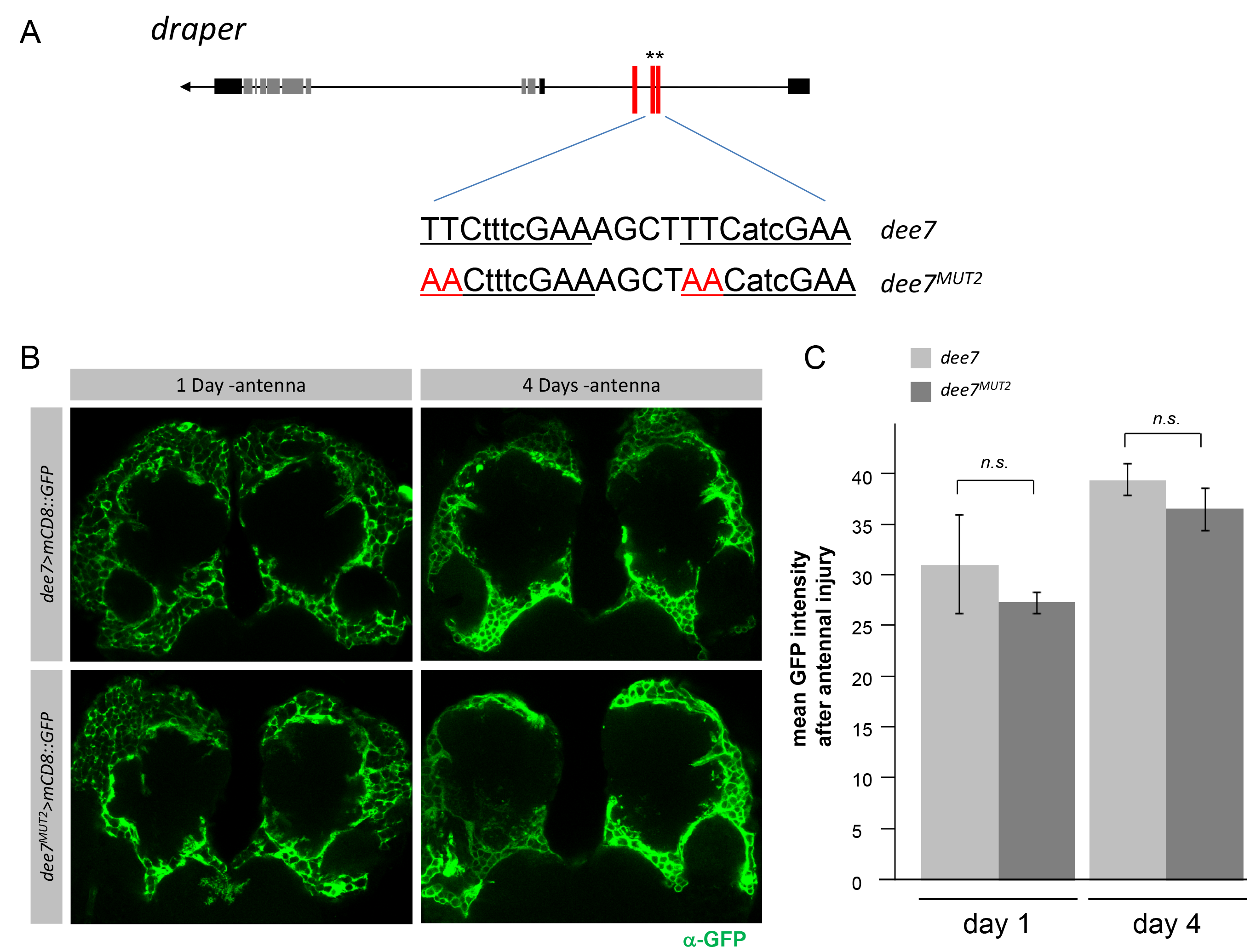

Supplement: Figure S2 — Simultaneous mutation of tandem Stat92e binding sites in the dee7 enhancer element does not affect dee7 activity after antennal ablation. (A) Schematic representation of the draper gene. Asterisks indicate location of the tandem Stat92E binding sites in the dee7 enhancer region. Underlined letters indicate potential Stat92E binding motifs. Red letters show nucleotides mutated in the dee7MUT2-Gal4 element. (B) Single slice confocal images of antennal lobe regions; dee7-Gal4 or dee7MUT2-Gal4 driving two copies of UAS-mCD8::GFP (dee7>2XmCD8::GFP, dee7MUT2>2XmCD8::GFP). One day after antennal ablation and four days after antennal ablation are shown. (C) Quantification for (B), p-values were calculated using Student's t test, n.s., not significant. Error bars represent SEM, n≥5 for all. (TIF) [file pbio.1001985.s002.tif]

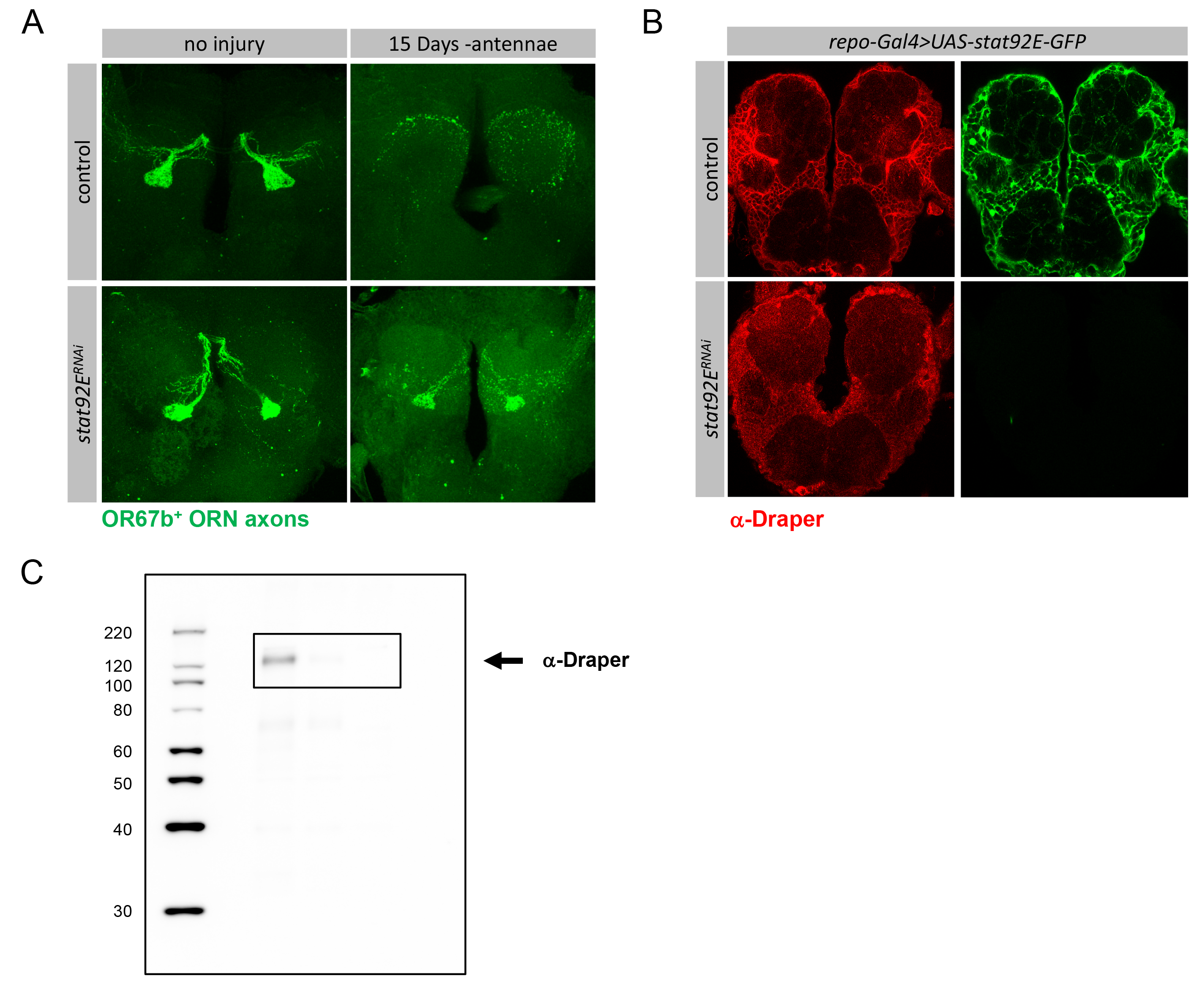

Supplement: Figure S3 — Glial specific knockdown of Stat92E inhibits axon clearance for at least 15 days after antennal ablation. (A) Z-stack confocal images of OR67b+ axons labeled with GFP in control (OR67b-GFP/+;repo-gal4/+) and stat92e RNAi (OR67b-GFP/UAS-stat92e RNAi;repo-gal4/+) backgrounds. Uninjured and 15 days after antennal ablation are shown. (B) Single slice confocal images of adult brains stained with α-Draper and α-GFP in control (UAS-stat92e-GFP/repo-gal4), and stat92e RNAi (UAS-stat92e RNAi/+; UAS-stat92e-GFP/repo-gal4) backgrounds. (C) Image of full Western blot for bands shown in Figure 3. (TIF) [file pbio.1001985.s003.tif]

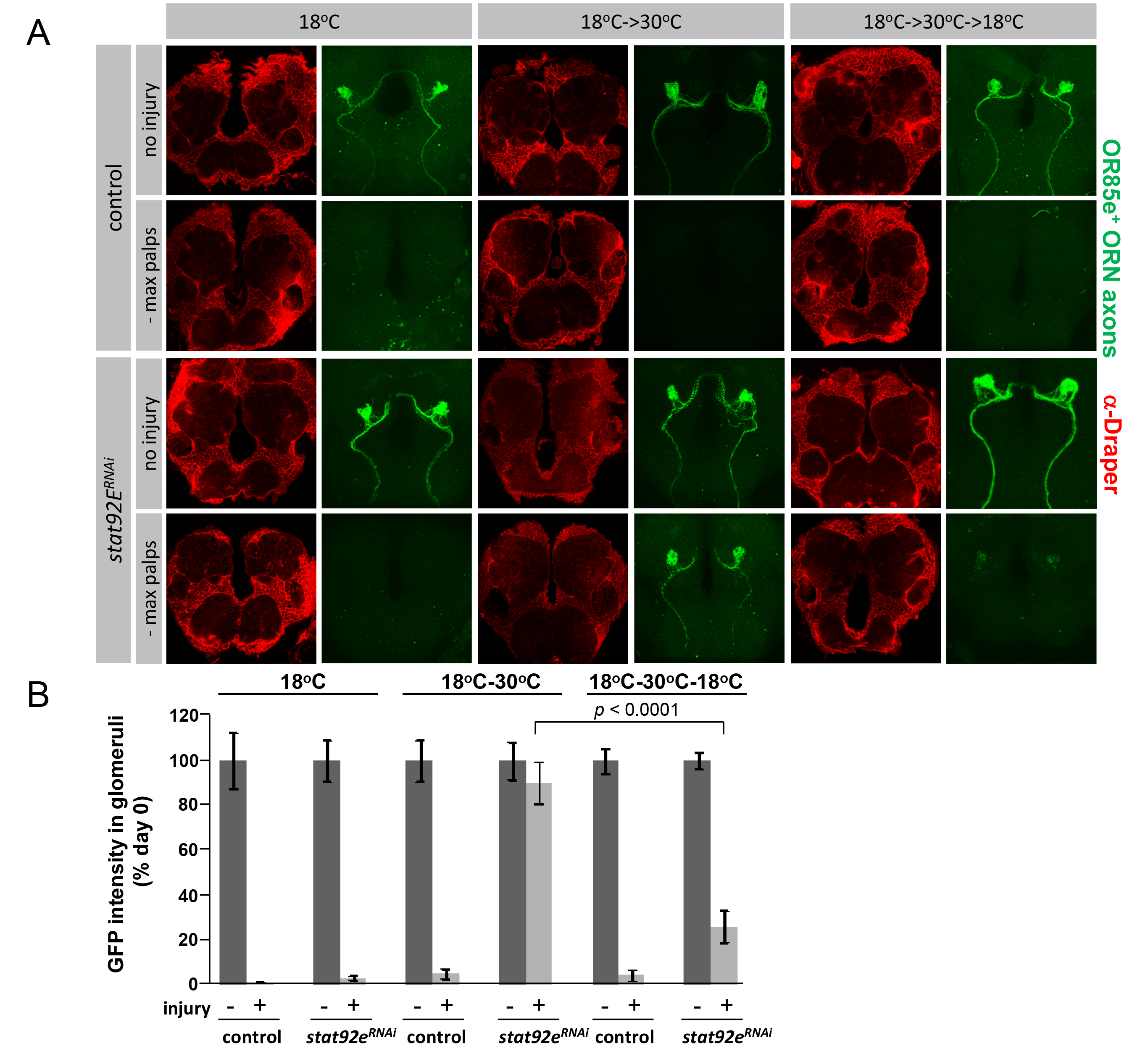

Supplement: Figure S4 — Stat92E is required in the adult brain for draper expression and glial engulfment function. (A) Z-stack confocal images of OR85e-GFP axons, single slice confocal images of Draper antibody stain; UAS-stat92e RNAi was driven in all glia using repo-Gal4 in a background containing the temperature sensitive Gal4 repressor, Gal80ts. Axons (OR85e-GFP, green) and Draper levels (red) are shown in control (OR85e-GFP,Gal80ts/+;repo-gal4/+) or stat92eRNAi knockdown (OR85e-GFP,Gal80ts/stat92eRNAi;repo-Gal4/+) animals. Temperature shifts were performed as follows: 18°C indicates that flies were raised and kept at 18°C throughout experiment. 18°C–30°C indicates that flies were raised at 18°C and then shifted to 30°C as adults for 7 days prior to and 5 days following maxillary palp ablation. 18°C–30°C–18°C indicates that flies were raised at 18°C, shifted to 30°C as adults for 7 days, and then shifted back to 18°C for 7 days prior to and 5 days following maxillary palp ablation. (B) Quantification of GFP intensities in 85e+ glomeruli from (A). Error bars represent SEM, n≥10. (TIF) [file pbio.1001985.s004.tif]

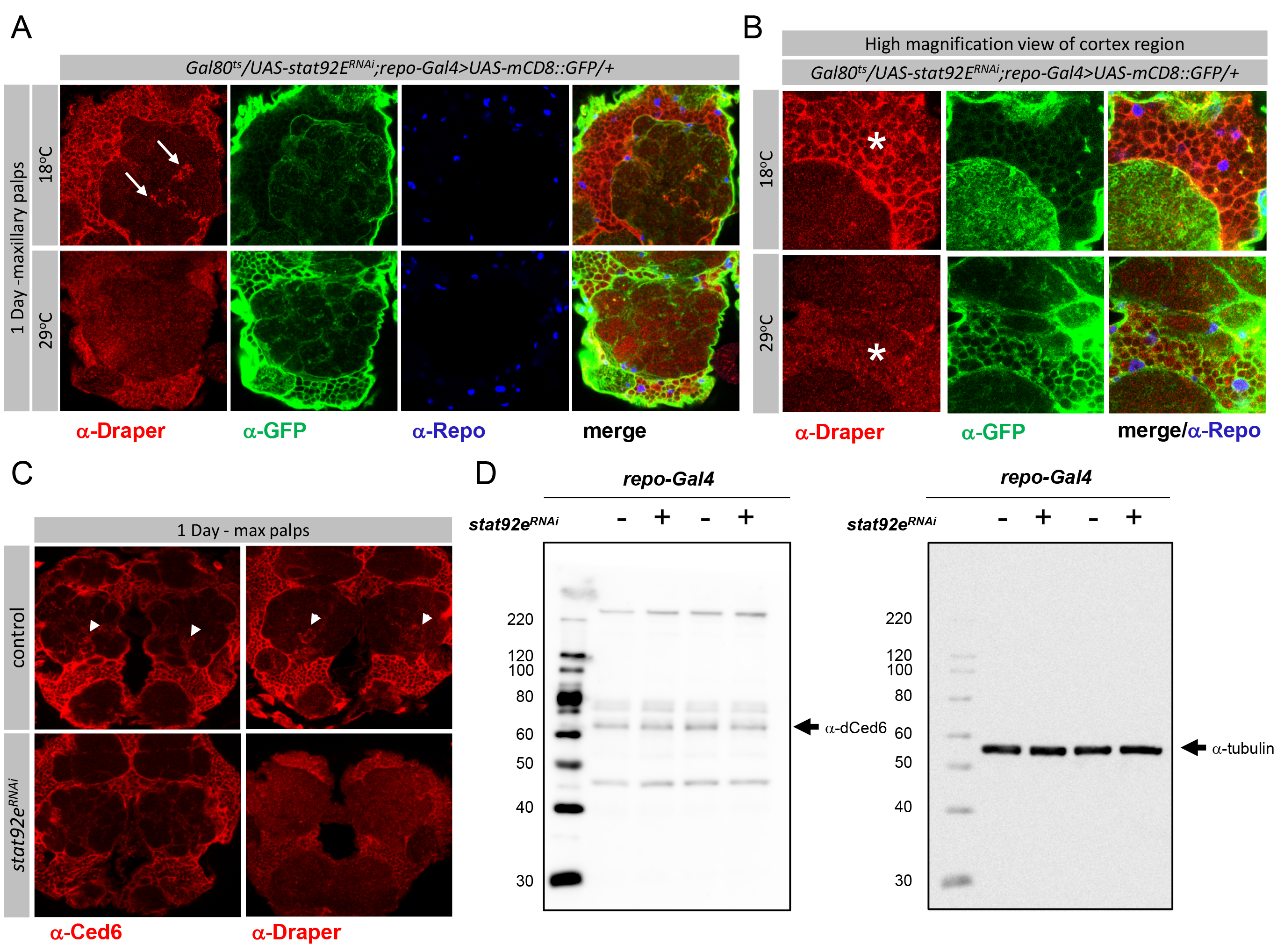

Supplement: Figure S5 — Glial specific knockdown of STAT92E does not disrupt glial morphology or result in loss of dCed-6 protein levels. (A) Single slice confocal images of adult brains of indicated genotype, (UAS-stat92eRNAi/+;repo-Gal4,UAS-mCD8::GFP/Gal80ts) one day after maxillary palp injury. Glial nuclei are labeled with anti-Repo (blue), glial membranes are labeled with GFP (green), and anti-Draper staining is shown (red). Flies were raised at 18°C and either kept at 18°C throughout the experiment or shifted to 29°C for 7 days prior to dissection. At the restrictive temperature of 18°C some repo-gal4 driven GFP is detectable but Draper staining looks grossly normal and glia are able to respond to injury one day after maxillary palp ablation. In flies shifted to the restrictive temperature, Draper staining is significantly reduced and no glial membranes are recruited to severed axons one day after maxillary palp injury. However, glial cell nuclei and membranes appear grossly normal in the stat92eRNAi flies. (B) Single slice confocal image of adult brain, high magnification view of cortex region of the brain. Asterisk indicates that while Draper is absent in the stat92eRNAi animals, glial morphology is normal. (C) Adult brains one day after maxillary palp removal, stained with α-dCed6 or α-Draper in control (OR85e-GFP,Gal80ts/+;repo-Gal4/+), and stat92eRNAi (OR85e-GFP,Gal80ts/stat92eRNAi;repo-Gal4/+) backgrounds. (D) α-Draper Western blot performed in duplicate on ∼3 adult central brain regions per lane in control (OR85e-GFP,Gal80ts/+;repo-Gal4/+), and stat92eRNAi (OR85e-GFP,Gal80ts/stat92eRNAi;repo-Gal4/+) backgrounds. α-Tubulin was used as a loading control. (TIF) [file pbio.1001985.s005.tif]

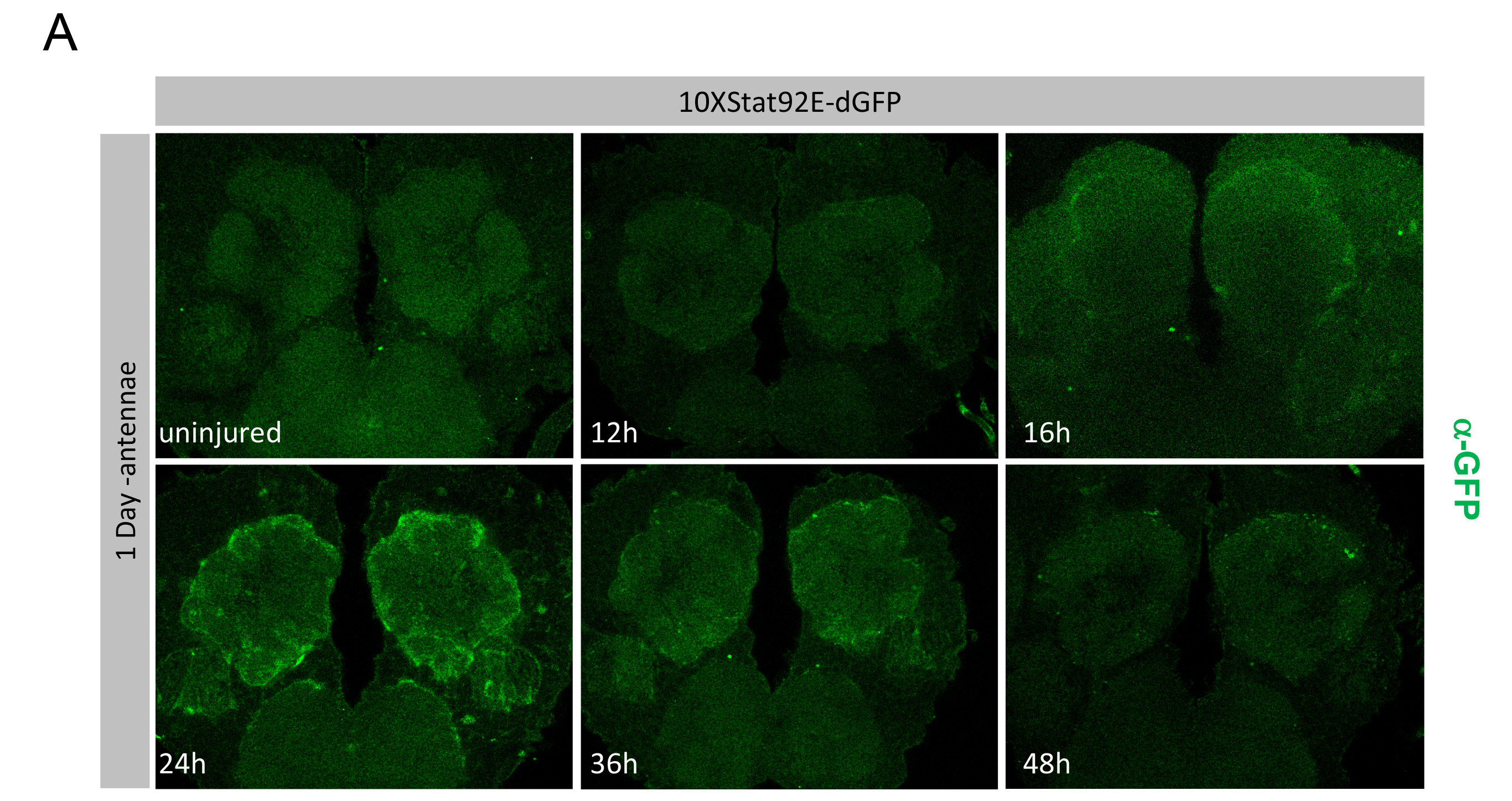

Supplement: Figure S6 — 48 hour time course showing activation of the 10XStat92E-dGFP reporter following antennal ablation. (A) Single slice confocal images of adult brains; 10XStat92E-dGFP reporter activity in an uninjured brain and at various timepoints after injury, 12 hours, 16 hours, 24 hours, 36 hours, and 48 hours. (TIF) [file pbio.1001985.s006.tif]

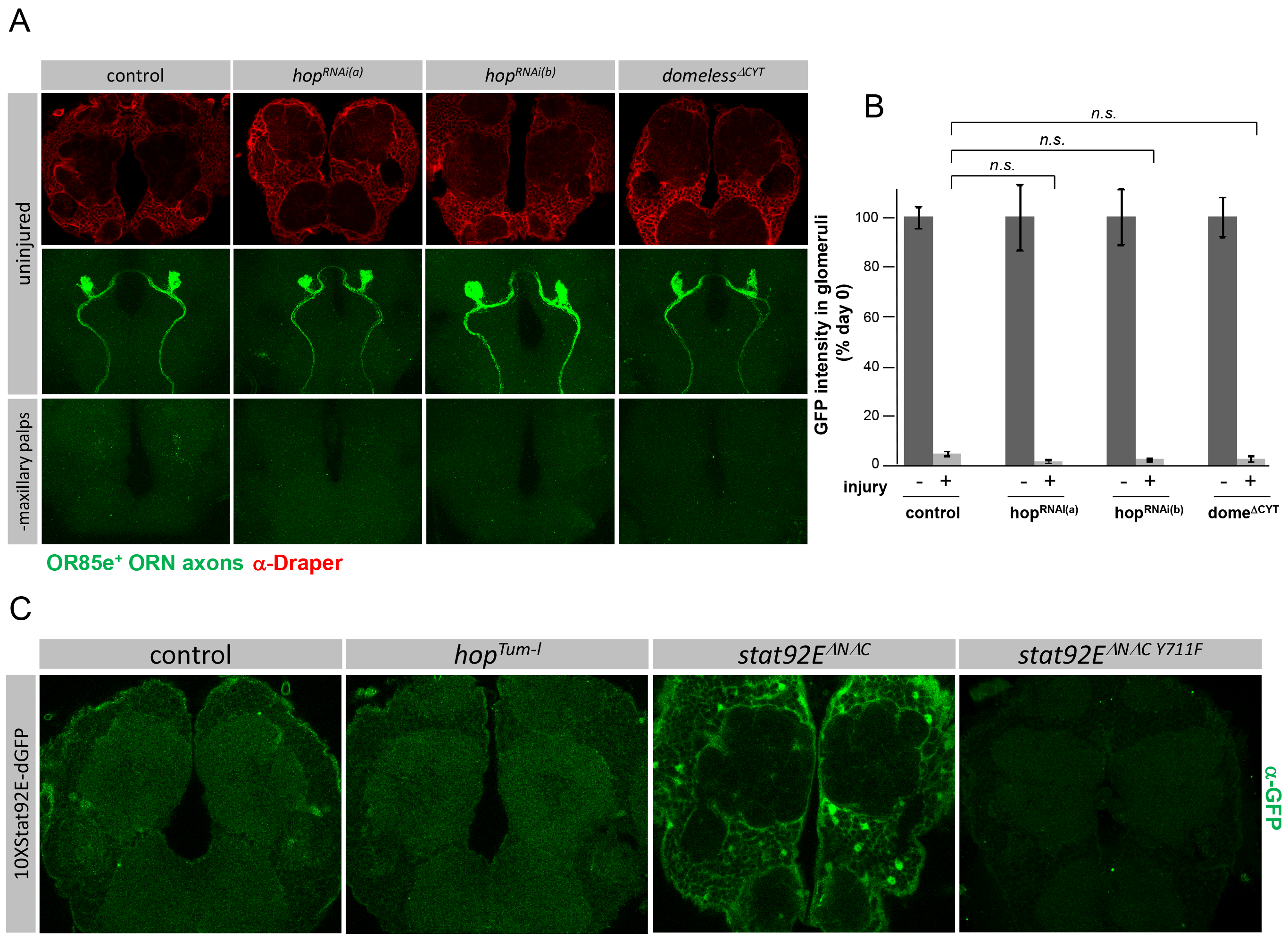

Supplement: Figure S7 — Glial activation of Stat92E activity after axotomy is not mediated by canonical JAK/STAT signaling. (A) repo-gal4 was used to drive two independent UAS-hop RNAi constructs and a UAS dominant negative domeless allele; single slice confocal images of Draper antibody stain and Z-stack confocal images of OR85e-GFP axons in control (OR85e-GFP/hop RNAi(a);repo-gal4/+), hopRNAi(a) (OR85e-GFP/+;repo-gal4/+) hopRNAi(b) (OR85e-GFP/UAS- hop RNAi(b);repo-gal4/+), domeless ΔCYT (OR85e-GFP/UAS-domeless ΔCYT;repo-gal4/+) (B) Quantification of data from (A). Error bars represent SEM, n≥10. (C) Single slice confocal images of indicated genotype in 10XSTATd-GFP background; control (10XStat92E-dGFP/+;repo-gal4/+), hopTUM(hopTUM/X;10XSTAT92E-dGFP/+;repo-gal4/+),stat92e ΔNΔC (10XSTAT92E-dGFP/+;repo-gal4/UAS- stat92e ΔNΔC), stat92e ΔNΔCY711F (10XStat92E-dGFP/stat92e ΔNΔCY711F;repo-gal4/+). (TIF) [file pbio.1001985.s007.tif]

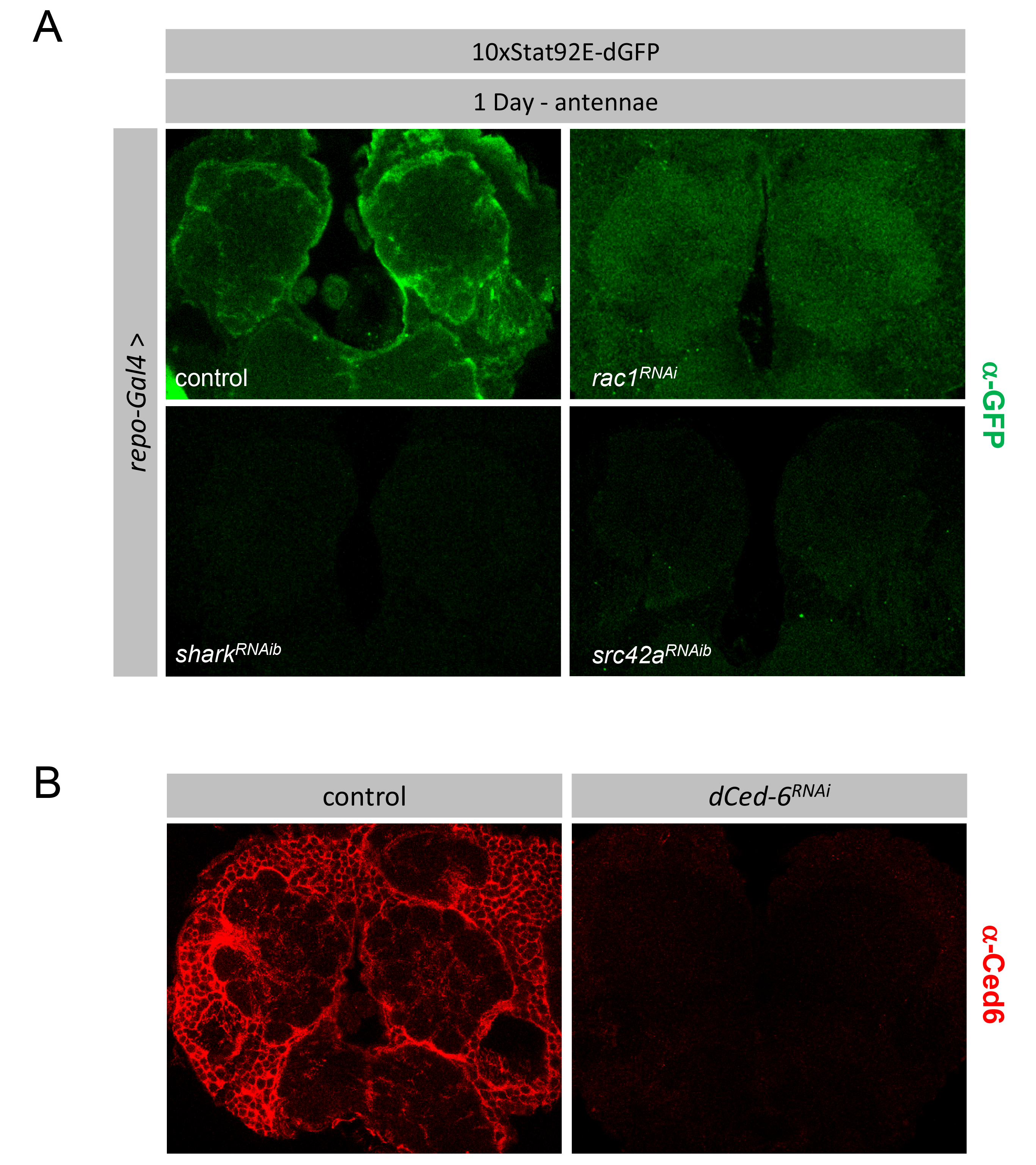

Supplement: Figure S8 — RNAi mediated knockdown of rac1, shark, and src42a results in loss of Stat92E transcriptional reporter activity after injury. (A) Glial-specific knockdown of components of the draper pathway in a 10XStat92E-dGFP reporter background one day after antennal ORN axotomy; control (10XStat92E-dGFP/+), sharkRNAi (sharkRNAi/10XStat92E-dGFP; repo-Gal4/+), rac1RNAi(rac1RNAi/+;10XStat92E-dGFP/+; repo-Gal4/+) and src42aRNAi (src42aRNAi/10XStat92E-dGFP; repo-Gal4/+). (B) Single slice confocal images of adult brains stained with α-dCed6 in control, (OR85e-GFP,Gal80ts/+;repo-Gal4/+), and d-ced6RNAi (OR85e-GFP,Gal80ts/d-ced6RNAi;repo-Gal4/+) backgrounds. (TIF) [file pbio.1001985.s008.tif]

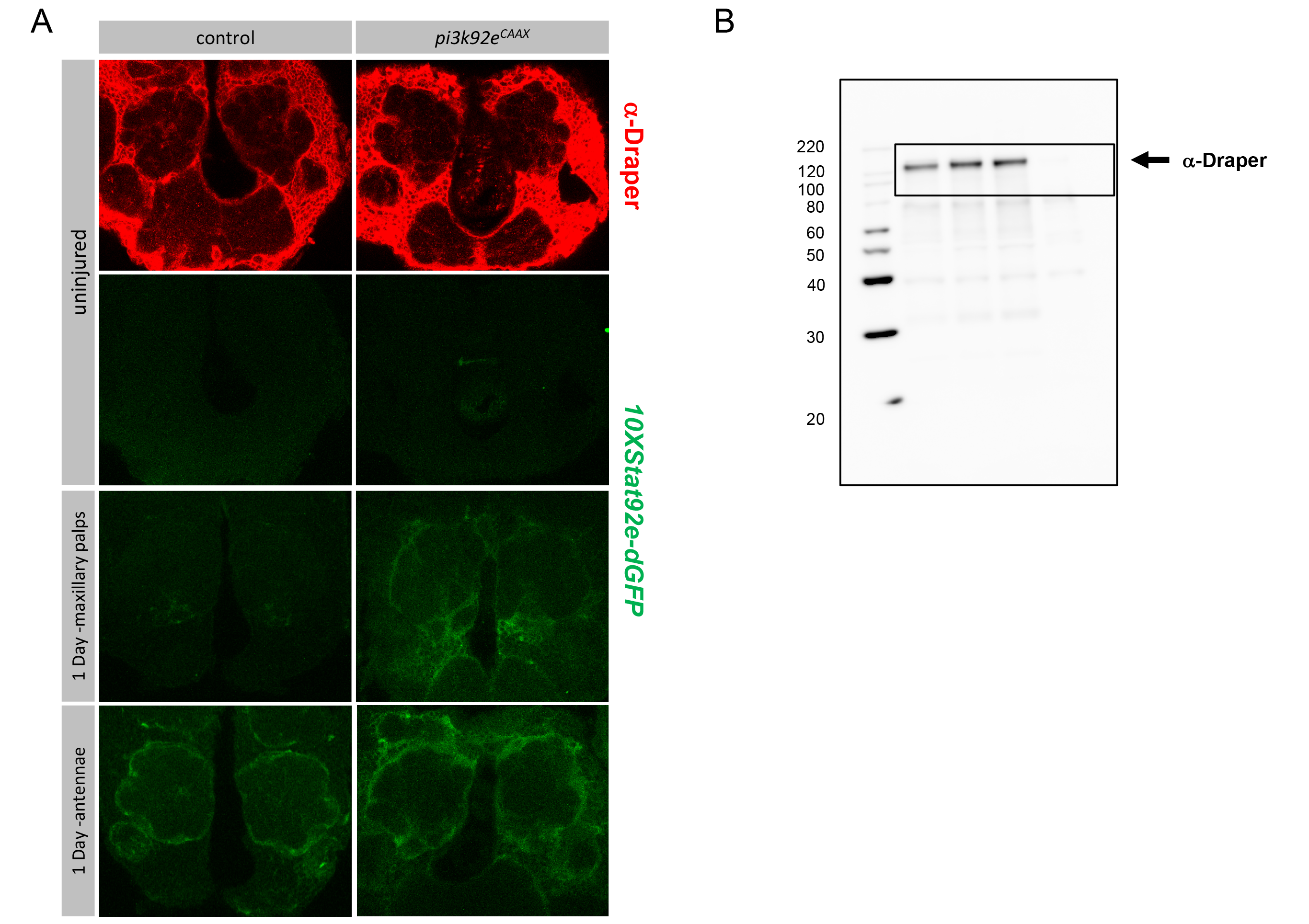

Supplement: Figure S9 — Constitutively active PI3K signaling does not activate the 10XStat-dGFP reporter. (A) Single slice confocal images of the adult brains stained with α-Draper or α-GFP in control (repo-Gal4/+; and 10XStat92E-dGFP/+;repo-Gal4/+), and pi3k92eCAAX (pi3k92eCAAX/+;repo-Gal4/+; and pi3k92eCAAX;10XStat92E-dGFP/+;repo-Gal4/+) backgrounds. Uninjured, one day after maxillary palp injury and one day after antennal injury are shown. (B) Image of full Western blot for bands shown in Figure 6. (TIF) [file pbio.1001985.s009.tif]
