# Supplementary material for: PI3K Signaling and Stat92E Converge to Modulate Glial Responsiveness to Axonal Injury
Source: PLoS Biol. 2014 Nov 4;12(11):e1001985. doi: 10.1371/journal.pbio.1001985 (PMC4219656; doi:10.1371/journal.pbio.1001985)
Supplement: Methods S1 — Supporting methods and references. (DOC) [file pbio.1001985.s024.doc]

**Supplementary Materials and Methods**

***Fly Strains and molecular biology***

(1) *UAS-pi3k92e^A2860C^*, Bloomington Stock 8288, (2) *UAS-stat92e-GFP* , (3) OR67b-GFP, (4) *UAS-hop^RNAi(a)^*, VDRC 40037, (5) *UAS-hop^RNAi(b)^*, VDRC 102830, (6) *UAS-domeless^ΔCYT^*  , (7) hop^Tum^ Bloomington Stock 8492, (8) *UASp-stat92e^ΔNΔC^* , (9) *UASp-stat92e^ΔNΔCY711F^* (DNA provided by E. Bach, transgenic flies generated by Best Gene using standard methods), (10) *UAS-dced6^RNAi^ .*

To generate the *dee7^MUT2^-Gal4* construct, a synthetic gene was generated by Biomatik Inc. (Wilmington, DE), which consists of a portion of the *dee7-Gal4* enhancer region with point mutations in two potential Stat92E binding sites. This synthetic gene was cloned into the *dee7*/TOPO construct using Bstz171 and Not1 restriction sites replacing the original portion of the *dee7* enhancer. Colonies were prepped using the Qiagen miniprep kit. The *dee7^MUT2^* enhancer fragment was then shuttled into the pBGUw vector using methods described above and transgenic flies were generated by Best Gene Inc. (Chino Hills, CA) using PhiC3 targeted integration.

***Temperature shift experiments***

To perform the temperature shift assay in Supplemental Figure 4 all flies were raised at the restrictive temperature of 18^o^C until eclosion. One group of flies was kept at 18^o^C throughout the experiment to ensure the Gal80ts was efficiently repressing the Gal4 driven RNAi. Another group of flies was shifted at the same time to the permissive temperature of 30^o^C for 7 days prior to maxillary palp ablation to allow the RNAi to turn on and they were kept at 30^o^C for an additional 5 days following maxillary palp ablation prior to dissection. The last group was shifted all at the same time to the permissive temperature of 30^o^C for 7 days to turn on the RNAi and then shifted back to the restrictive temperature of 18^o^C for 7 days to turn off the RNAi prior to maxillary palp ablation. They were kept at 18^o^C for 5 days following maxillary palp ablation and the brains were dissected.

***Western Blot***

*Drosophila* brains of the indicated genotype were dissected in PBS and homogenized in SDS loading buffer (60 mM Tris pH 6.8, 10% glycerol, 2% SDS, 1% -mercaptoethanol, 0.01% bromophenol blue). For Western analysis, samples containing approximately 3 brains were loaded onto 10% SDS-PAGE gels (BioRad), transferred to nitrocellulose membranes (BioRad), and probed with rat α-dCed-6 antibody at 1:1000 diluted in PBS/0.01% Tween-20/5% BSA . Blots were incubated overnight at 4 degrees, washed several times in PBS/0.01% Tween-20 and probed with the appropriate HRP conjugated secondary antibody for 2 hours at room temperature. Additional washes were performed and the blot was developed using chemiluminescence (Amersham ECL Plus), and detected with a Fujifilm Luminescent Imager. The protein blot was stripped with mild stripping buffer (0.2M glycine, 0.1% sodium dodecyl sulfate, 1% Tween, pH 2.2) at room temperature followed by washes in 1XPBS and 1XPBS + 0.01% Tween-20 and then reprobed with mouse α-tubulin (Sigma), 1:1000.

1. Karsten P, Plischke I, Perrimon N, Zeidler MP (2006) Mutational analysis reveals separable DNA binding and trans-activation of Drosophila STAT92E. Cell Signal 18: 819-829.

2. Silver DL, Montell DJ (2001) Paracrine signaling through the JAK/STAT pathway activates invasive behavior of ovarian epithelial cells in *Drosophila*. Cell 107: 831-841.

3. Ekas LA, Cardozo TJ, Flaherty MS, McMillan EA, Gonsalves FC, et al. (2010) Characterization of a dominant-active STAT that promotes tumorigenesis in Drosophila. Dev Biol 344: 621-636.

4. Awasaki T, Tatsumi R, Takahashi K, Arai K, Nakanishi Y, et al. (2006) Essential role of the apoptotic cell engulfment genes draper and ced-6 in programmed axon pruning during Drosophila metamorphosis. Neuron 50: 855-867.
